# Expansion of the functional genomics GRACE library reveals genes relevant for temperature-dependent fitness in *Candida albicans*

**DOI:** 10.1371/journal.pbio.3003409

**Published:** 2025-10-17

**Authors:** Ci Fu, Emily H. Xiong, Livia Kupczok, Linda S. Archambault, Timothy R. W. Wang, Caitlin Holleran, Duncan Carruthers-Lay, Ting Xuan Zhuang, Sofia Marcoccia, Haoyang Zhang, Kevin Chen, Daniel Anderson, Bonnie Yiu, Zhongle Liu, Lydia Herzel, Nicole Robbins, Leah E. Cowen

**Affiliations:** 1 Department of Molecular Genetics, University of Toronto, Toronto, Ontario, Canada; 2 Institute of Chemistry and Biochemistry, Freie Universität Berlin, Berlin, Germany; University of Georgia, UNITED STATES OF AMERICA

## Abstract

A small percentage of species in the fungal kingdom can cause devastating infections in humans, with *Candida albicans* reigning as a leading cause of systemic disease. One of the key virulence phenotypes for pathogenic fungi is the ability to survive at host body temperature; however, a comprehensive understanding of the mechanisms that orchestrate thermal adaptation in fungi remains incomplete. In this study, we expand the largest functional genomics resource in *C. albicans,* reaching 71.3% coverage of the entire genome, and perform screens under six different temperatures to identify genes important for temperature-dependent fitness. We describe the function of genes involved in translation (*GAR1*), splicing (*C1_11680C* or *YSF3*), and cell cycle progression (*C6_00110C* or *RHT1*) in enabling fungal survival at both low and high temperatures. Through experimental evolution, we also show that *C. albicans* can rapidly overcome deleterious mutations and adapt to extreme temperature environments. Overall, our study highlights the transformative potential of genome-wide functional genomics to uncover critical vulnerabilities in pathogenic fungi.

## Abstract 2

*Candida albicans* is a leading opportunistic fungal pathogen of humans. This study expands the GRACE library, the largest functional genomics resource in *C. albicans* , and identifies genes important for temperature-dependent fitness, highlighting the transformative potential of functional genomics to uncover vulnerabilities in pathogenic fungi.

## Introduction

Human fungal pathogens pose major threats to human health, infecting over 6.55 million people with life-threatening conditions and directly contributing to 2.55 million deaths annually [1]. Among the estimated 5 million fungal species, only a few hundred can cause infections in humans [2]. One of the major barriers restricting fungal species from becoming human pathogens is the ability to grow at human physiological temperature [3]. However, climate change has been implicated in enabling thermal adaptation in fungi, facilitating geographical spread of endemic fungal diseases, and expanding the human fungal pathogen repertoire [4,5]. For example, higher global temperatures have been suggested to promote the spread of the fungal pathogen *Cryptococcus deuterogattii*, which is more thermotolerant than other species in the same *Cryptococcus gattii* species complex [6,7]. Another emerging fungal pathogen, *Candida auris*, first identified from an ear infection in 2009 in Japan, is now a significant public health concern causing nosocomial outbreaks in hospitals globally [8,9]. Importantly, the global emergence of *C. auris* as a human pathogen is hypothesized to stem from adaptations driven by climate change, allowing it to transition from historically environmental niches to human hosts [10]. Thus, understanding how fungi adapt to extreme temperatures is critical to understand how these species spread and cause disease in human hosts.

Deviation from optimal growth temperatures at times induces a temperature shock that disrupts protein homeostasis, challenges cellular physiological state, and jeopardizes fungal survival [11]. In response to temperature upshifts, fungal cells employ a conserved heat shock response, involving reprogramming of gene expression to repress protein biosynthesis and up-regulating cytoprotective heat shock protein-encoding genes. Two of the key players in that response are heat shock protein 90 (Hsp90) and heat shock transcription factor 1 (Hsf1), which are conserved from yeasts to humans. In the commensal fungal pathogen *Candida albicans*, the Hsf1-Hsp90 autoregulatory circuit governs the cellular transcriptional response to heat stress [12]. Upon transient heat shock, *C. albicans* cells rapidly up-regulate over 12% of the genome in an Hsp90-dependent manner with gene functions enriched in the unfolded protein response, proteasome/ubiquitination, oxidative stress response, cell cycle, biofilm formation, and pathogenesis [13]. Hsp90 orchestrates this transcriptional response by modulating chromatin nucleosome positioning enabling transcriptional regulation by Hsf1 [13]. Additional cellular stress response pathways are implicated in tolerance to extreme temperatures as well. In the opportunistic fungal pathogen *Cryptococcus neoformans*, calcineurin, a conserved calcium-calmodulin-dependent serine/threonine-specific phosphatase modulates heat stress to enable cell survival, and the cold stress response is regulated by the phosphatase Gpp2, a target of the Hog pathway [14–17]. Furthermore, biofilms, or surface-associated communities, have been shown to enhance fungal tolerance to both heat and cold stresses [18]. Due to the pleiotropic effects of these temperature shock defense mechanisms, heat/cold stress adaptation is often accompanied by broader physiological changes, leading to alterations in the cell cycle, transcription, translation, and metabolic processes, which can enhance the potential for fungal pathogenesis [4,11].

With the growing threat of fungal pathogens to human health, the antifungal arsenal remains limited, consisting of only three major classes of antifungal drugs (polyenes, azoles, and echinocandins) targeting essential functions [19]. Unfortunately, each of these antifungal classes is limited by significant host toxicity issues, limited spectrum of activity, and/or the frequent development of resistance [20]. Moreover, heat stress has been shown to induce aneuploidy formation in all major fungal pathogens, including *C. albicans,* to enable antifungal drug tolerance and resistance [21]. Amid the threat of climate change, fungal pathogens are primed to gain the upper hand in the ongoing antifungal arms race, highlighting the urgency in understanding fungal thermal adaptation.

In *C. albicans,* genetic mutant libraries have enabled large-scale functional genomic screens to identify and characterize genes that govern fungal survival, virulence, and pathogenesis [22–27]. One of the largest functional genomic resources in *C. albicans* is the Gene Replacement and Conditional Expression (GRACE) collection consisting of heterozygous deletion mutants, where the expression of the remaining wild-type allele is regulated by a doxycycline (DOX)-repressible promoter [23]. Upon its original release, this collection consisted of mutants representing 2,326 genes or ~37% of the 6,198 genes in the *C. albicans* genome [23]. Genomic coverage increased to 48% with the release of an expanded set of mutants representing 866 genes (GRACEv2) [25]. However, despite the instrumental role this collection has made in furthering our understanding of *C. albicans* biology [27], over half of the genome has yet to be investigated to identify genes required for important biological traits, including growth at extreme temperatures.

Here, we introduce the expansion of GRACE mutants, bringing the cumulative coverage of the GRACE library to 71.3% of the *C. albicans* genome. Screening of the expanded GRACE library at multiple temperatures identified hundreds of genes that are important for fitness, many of which were selectively required for growth at extreme temperatures. Through the integration of phenotypic clustering, structural similarity analyses, and experimental evolution, we described the function of genes involved in translation (*GAR1*), splicing (*C1_11680C* or *YSF3*), and cell cycle progression (*C6_00110C* or *RHT1*). Furthermore, through experimental evolution, we showed that *C. albicans* can rapidly overcome deleterious mutations via diverse mechanisms in response to high temperature. Overall, this work significantly expands a critical functional genomics resource in *C. albicans* and characterizes the function of unknown genes with temperature-dependent importance for fitness in this human fungal pathogen.

## Results

### Screening of the expanded GRACE library identifies functionally enriched gene clusters with temperature-dependent importance for fitness

**Fig 1:**
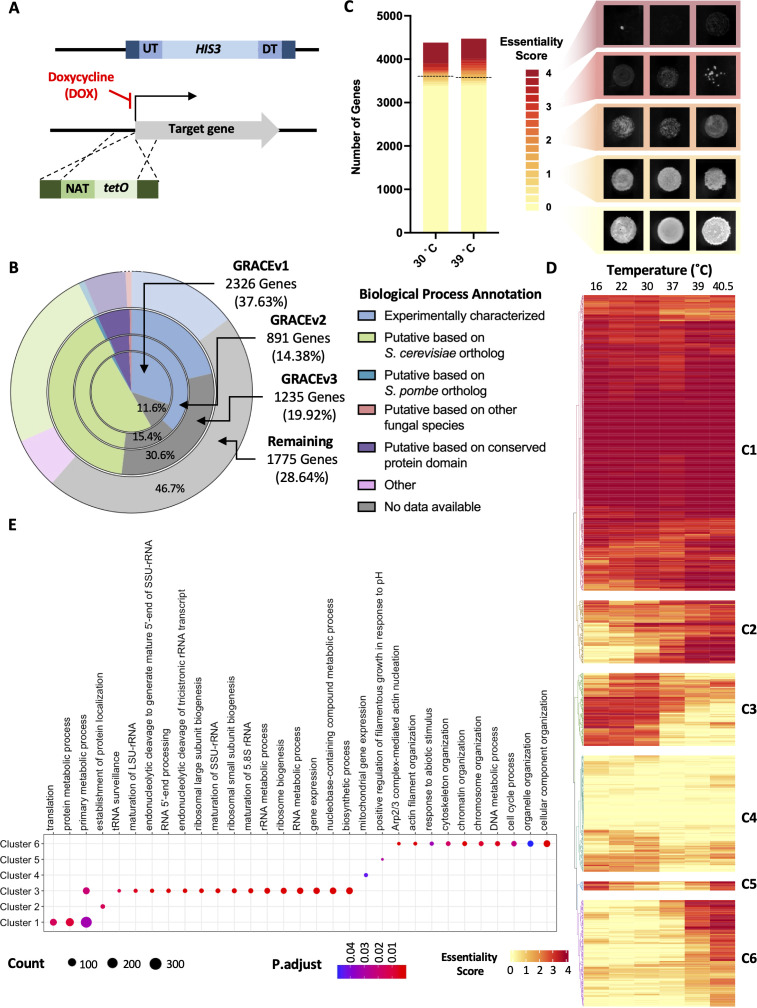
Screening of expanded GRACE mutant collection at six temperatures reveals distinct functional enrichment in genes clustered by fitness profile. **A)** Schematic of GRACE mutant construction. **B)** Summary of GRACE mutant collection expansion based on manual curation of biological process gene ontology annotation on *Candida* Genome Database (CGD). Pie chart with four concentric rings visualizes the relative proportion of genes from various versions of the GRACE collection with biological process descriptions completely unannotated or annotated based on: experimentally characterization, *S. cerevisiae* ortholog, *S. pombe* ortholog, orthologs in other fungal species, conserved protein domains, or unannotated; see Materials and methods for full details. **C)** Screening results of GRACE v1, v2, and v3 at 30 and 39 °C. Colors in stacked bar graph represent fitness scores of mutants, with a score of 4 (red) representing no growth and 0 (yellow) representing growth comparable to wild type. Representative images of mutants corresponding to scores are shown. Dotted line denotes cutoff for genes with fitness score > 1 selected for further screening. **D)** Heatmap of genes ordered by hierarchical clustering of essentiality scores across screening temperatures. A score of 4 (red) represents no growth and 0 (yellow) represents growth comparable to wild type. **E)** Dot plot representing gene ontology (GO) enrichment analysis of genes in clusters from panel D generated by hierarchical clustering. Dot size represents the number of genes identified for the given GO term and the *P*-value adjusted by false discovery rate is shown on a blue to red scale with red being the most significant. The data underlying this Figure can be found in S1 Data.

Despite the clinical importance of **C. albicans*,* our understanding of this fungal pathogen remains incomplete due to a lack of comprehensive mutant collections to interrogate gene function. Thus, we first sought to expand the GRACE collection to improve genome coverage. Specifically, *C. albicans* heterozygous deletion mutants marked with strain-specific molecular barcodes [23] were transformed with a DNA construct consisting of a nourseothricin (NAT)-resistance marker, a DOX-repressible promoter, and homologous sequences to drive homologous recombination immediately upstream of the start codon of the target gene of the remaining allele (Fig 1A). Through this approach, we generated 1,240 novel GRACE strains (GRACEv3) representing 1,228 genes absent from the previous two iterations, expanding genome coverage of this collection from ~48% to ~71% (Fig 1B). For those strains present in the GRACE collection, genes were grouped into broad categories based on manual curation of their annotation status from the *Candida* Genome Database (CGD) in the gene ontology category of “biological process” [28]. Specifically, genes were determined to have been annotated (i) directly with experimental characterization in *C. albicans*, (ii) indirectly with conservation in sequence and/or protein domains to other fungal species, or (iii) unannotated based on evidence codes denoting the type of experiments or prediction data presented to support functional annotations. Construction of GRACEv1 and v2 libraries was guided by orthologous gene functions covering all major biological processes in *Saccharomyces cerevisiae*. Thus, unannotated genes only make up 11.6% and 15.4% of genes in these two iterations [23,25] (Figs 1B and S1A). In contrast, the construction of GRACEv3 library was not guided by gene functions in *S. cerevisiae*, resulting in the inclusion of 375 genes (30.6%) lacking functional annotations (Figs 1B and S1A). The remaining ~50% of unannotated genes in the *C. albicans* genome will benefit from continued efforts of library expansion.

With our expanded *C. albicans* GRACEv3 collection, we next wanted to identify genes that were important for fitness at 30 °C, as previously performed for GRACEv1 and v2 mutants [25]. To do so, the arrayed GRACEv3 library was grown in liquid culture with a high concentration (100 μg/mL) of DOX before transferring onto solid medium containing 100 μg/mL DOX in technical duplicate to ensure maximum transcriptional repression. Plates were incubated at 30 °C for 48 h. Colony images were scored independently by two researchers from 0 to 4, with 4 representing no growth and 0 representing growth comparable to wild type [25] (Figs 1C and S1B). Given the importance of thermotolerance for the success of *C. albicans* as a human fungal pathogen, we also screened the entire GRACE collection (v1, v2, and v3) for growth at elevated temperature (39 °C) using an analogous approach. Screening results for the GRACEv3 collection at 30 °C were compiled alongside previous results from the v1 and v2 collections [25] and compared to those obtained at 39 °C. This revealed 793 and 887 genes that were important for *C. albicans* fitness at 30 and 39 °C, respectively, with 734 genes that were important at both temperatures (essentiality score >1, Figs 1C and S1C).

To further interrogate the role of temperature in *C. albicans* fitness, we arrayed 947 strains that showed moderate to severe growth defects (average score > 1 in the presence of DOX) in our initial screen (Fig 1C). To avoid preselection for genes important for fitness during adaptation to DOX suppression, mutants were grown overnight in liquid culture in the absence or presence of 0.5 µg/mL DOX at 30 °C. Mutants were then spotted onto agar plates in the presence and absence of 100 µg/mL DOX and incubated at six different temperatures (16, 22, 30, 37, 39, and 40.5 °C). At the respective timepoints, plates were imaged, and growth of mutants was scored independently by three individuals and averaged to obtain a final growth score for each mutant (S1D Fig and S1 Data). Compared to the 887 strains that were annotated with an essentiality score >1 at 39 °C in the initial screen, 648 strains scored >1 at 39 °C in the secondary screen, suggesting lower DOX pretreatment enabled a more fine-tuned assessment of genes important for fitness at different temperatures (S1 Data). Mutants were then organized by hierarchical clustering of growth scores across screened temperatures to identify clusters of genes with minimal to moderate importance for growth (cluster 4), as well as temperature-independent (cluster 1), heat-sensitive (clusters 2 and 6), cold-sensitive (cluster 3), and extreme temperature-sensitive (cluster 5) phenotypes (Fig 1D). To identify functional enrichment within each cluster, gene ontology (GO) analysis was performed using the 947 genes screened as the background (Fig 1E). As expected, mutants displaying temperature-independent essentiality (cluster 1) were enriched for core biological processes like ‘translation’ and ‘protein metabolic process’, which are indispensable for survival. Cold tolerance genes (cluster 3) showed significant enrichment of various RNA metabolism and regulatory-related processes. Additionally, the GO term “filamentation in response to pH” was enriched in cluster 5, which consisted of genes essential for survival at both low and high temperatures. Finally, genes grouped together based on essentiality at extreme high temperatures (cluster 6) were enriched for functions related to cell cycle, filamentation, and stress response, biological processes previously shown to be important for thermotolerance [13,29]. Overall, this highlights that genes involved in similar biological processes often cluster together based on growth phenotypes at different temperatures.

### Gar1 modulates translation activity enabling fungal survival

**Fig 2:**
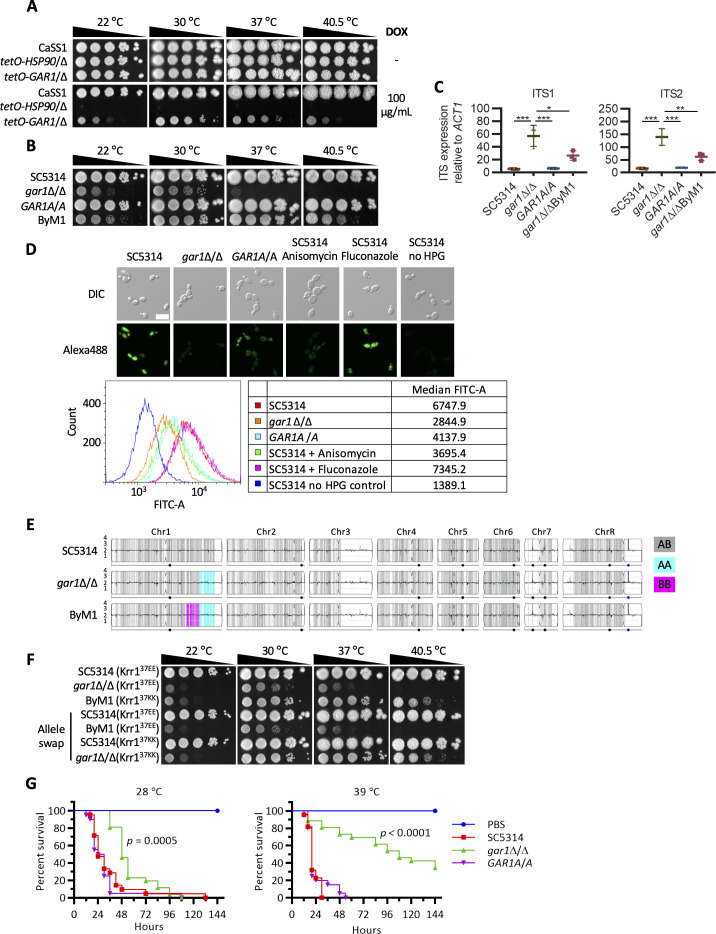
*Candida albicans GAR1* is important for rRNA maturation, translation, and virulence. **A)** Strains were grown overnight in YPD with or without 0.05 µg/mL DOX and spotted in 10-fold dilutions starting from an OD_600_ of 0.8 onto YPD with or without 100 µg/mL DOX. Plates were incubated at indicated temperatures and imaged after 5 days. **B)** Spotting assay was conducted as described in A. *GAR1A*/*A* represents the *gar1*Δ/Δ strain which was complemented with both A alleles. **C)** Cells were sub-cultured and grown to log phase at 30 °C before RNA extraction and cDNA synthesis. Total levels for the internal transcribed spacers 1 (ITS1) and 2 (ITS2) were measured by reverse transcriptase quantitative PCR (RT-qPCR) and normalized to *ACT1*. Error bars represent the mean ± SD for biological triplicates (* *p* ≤ 0.05, ** *p* ≤ 0.01, *** *p* ≤ 0.001, One-way ANOVA Bonferroni’s correction). **D)** Overnight cultures were sub-cultured to an OD_600_ of 0.1 in SD medium and grown at 30 °C for 4 h. Wild-type cells treated with 100 µg/mL anisomycin and 4 µg/mL fluconazole for 10 min were used as translation inhibitor and non-translation inhibitor controls, respectively. A l-homopropargylglycine (HPG) alkyne methionine analog was added, and cells were fixed. An azide fluorophore was added, and cells were imaged on the GFP channel to detect translation activity. Wild-type cells without HPG were used as no-signal control. Scale bar represents 10 µm. Fluorescence intensity was analyzed by flow cytometry using the fluorescein isothiocyanate (FITC) channel and plotted as a histogram. The median FITC signal intensity for each sample is listed in the table. **E)** Y-MAP plot visualizing chromosome copy number and loss of heterozygosity events from WGS analysis. Haplotypes relative to the reference genome SC5314 are color-coded (see legend). **F)** Spotting assay was conducted as described in A, except the plates were imaged after 3 days. **G)** Strains were inoculated at 1 × 10^6^ cells per larva with 20 larvae per group. A mock infection using PBS served as a control. Larvae were incubated at indicated temperatures and survival was monitored. Log rank (Mantel-Cox) test was performed to test significance between wild type and *gar1*Δ/Δ groups. The data underlying Fig 2C, 2D and 2G can be found in S1 Data.

The findings from our functional genomic screen revealed a distinct set of genes required for growth, particularly at elevated temperature, many of which were uncharacterized or poorly characterized based on *S. cerevisiae* orthologs. One of these genes from cluster 6 was the putative H/ACA snoRNP pseudouridylation complex protein, Gar1. Pseudouridylation is the most abundant RNA modification in noncoding RNAs in all living organisms and plays essential roles in transfer RNA and ribosomal RNA (rRNA) functions [30]. To verify the importance of *GAR1* for *C. albicans* growth at elevated temperature, we performed spot dilution assays with the *tetO-GAR1*/Δ strain in the absence and presence of DOX and observed a severe growth defect at all temperatures tested upon DOX-mediated transcriptional repression, particularly at 22 and 40.5 °C (Figs 2A and S2A). To confirm the importance of *GAR1* for *C. albicans* fitness, a homozygous deletion mutant was constructed and assessed for growth at different temperatures. Similar to the GRACE strain, spot dilution assay confirmed the importance of this gene for *C. albicans* fitness, particularly at 40.5 °C where no growth was observed for the deletion mutant. Complementation of the deletion mutant with two wild-type alleles (*GAR1A*/*A)* resulted in growth phenotypes similar to wild type (Fig 2B).

In *S. cerevisiae*, the box H/ACA ribonucleoprotein complex converts uridine into pseudouridine in rRNAs and consists of four core proteins (Cbf5, Nop10, Nhp2, and Gar1), one RNA-binding protein (Naf1), and one chaperone (Shq1) [31–33]. Spot dilution assays confirmed the importance of the *C. albicans *core genes for growth at all temperatures. In contrast, the chaperone *SHQ1* had the least impact on fitness across all temperatures compared to other genes of the box H/ACA ribonucleoprotein complex (S2A and S3 Figs). These observations suggest Gar1 may be partially dispensable for complex function under optimal conditions, but becomes absolutely required at extreme temperatures.

The box H/ACA ribonucleoprotein complex is important for rRNA pseudouridylation modifications and maturation in *S. cerevisiae* [33,34]. To investigate the role of Gar1 in *C. albicans*, we quantified the presence of precursor internal transcribed spacers (ITS1 and ITS2) by qRT-PCR. During rRNA maturation, ITS1 and ITS2 are cleaved from precursor rRNAs to form mature 18S, 5.8S, and 25S rRNAs [35]. Deletion of *GAR1* resulted in significant increases in ITS1 and ITS2 relative to wild-type and complemented strains (Fig 2C), similar to what was observed upon depletion of the core H/ACA ribonucleoprotein *CBF5* (S2A and S2B Fig), supporting a role of Gar1 in rRNA maturation (Fig 2C). Defects in rRNA maturation ultimately result in impaired translation [36]. Hence, we performed a fluorescence-based assay to monitor translation activity in the presence or absence of *GAR1* [37]. An alkynylated methionine analog, l-homopropargylglycine (HPG), was used to label newly synthesized proteins, and a click reaction of the alkyne with a fluorescent azide provided a fluorescent readout of the translation activity. Treatment of wild-type cells with the translation inhibitor anisomycin or deletion of *GAR1* reduced the fluorescent signal compared to wild type or the complemented control. Wild-type cells treated with the non-translation inhibitor fluconazole did not show reduced fluorescent signal, confirming the reduction in fluorescence intensity was not simply due to reduced cell growth (Fig 2D).

To further elucidate the molecular mechanism underlying Gar1’s role in temperature-dependent essentiality, we devised a selection strategy to identify mutants with a restored ability to grow at elevated temperature. To this end, we plated 10^8^ *gar1*Δ/Δ cells on agar plates, incubated the cells at 42 °C for 14 days, and successfully isolated one bypass mutant (ByM1) that was able to grow at 40.5 °C (Figs 2B and S4A) and partially restored ITS1 and ITS2 to wild-type-like levels (Fig 2C). Whole genome sequencing analysis of the evolved mutant showed no aneuploidy or copy number variation in comparison to the reference SC5314 genome or compared to the *gar1* mutant from which it was derived (Fig 2E). Variant calling identified a mutation in *KRR1* in the LOH region (cyan) on Chr1 at position 109, resulting in an amino acid substitution at position 37 from glutamic acid (E) to lysine (K). *KRR1* encodes a nucleolar protein functioning in the 90S pre-ribosome facilitating pre-rRNA cleavage during rRNA maturation [38]. To confirm this substitution was sufficient for the restored growth observed at elevated temperature, we replaced both *KRR1* alleles in the *gar1* mutant with those identified in ByM1 (Krr1^E37K^) and confirmed this strain was able to grow at high temperature (Fig 2F). To confirm this substitution was necessary for the growth at elevated temperatures, we replaced both *KRR1* alleles in ByM1 with those from the parental *gar1* mutant (Krr1^K37E^), which resulted in a loss of growth at 40.5 °C (Fig 2F).

Finally, we evaluated whether the temperature-dependent essentiality of *GAR1* plays a role in virulence using the wax moth *Galleria mellonella* model at both 28 and 39 °C [27]. This invertebrate model has successfully been used to study *C. albicans* pathogenesis and highlight those genes important for *C. albicans* virulence in a temperature-dependent manner [27,39]. We infected larvae by proleg injection with wild-type, *gar1*Δ/Δ, or complemented strains and incubated the *G. mellonella* at 28 or 39 °C. Deletion of *GAR1* significantly attenuated virulence at both temperatures compared to wild-type and the complemented strain with a greater significance at 39 °C, confirming the importance of Gar1 in *C. albicans* pathogenesis in vivo, especially at human febrile temperature (Fig 2G). Overall, these experiments support the role of Gar1 in thermal tolerance and highlight the increased dependency on translation and rRNA processing at elevated temperature for *C. albicans*.

### *C1_11680C* encodes Ysf3 and modulates splicing activity

**Fig 3:**
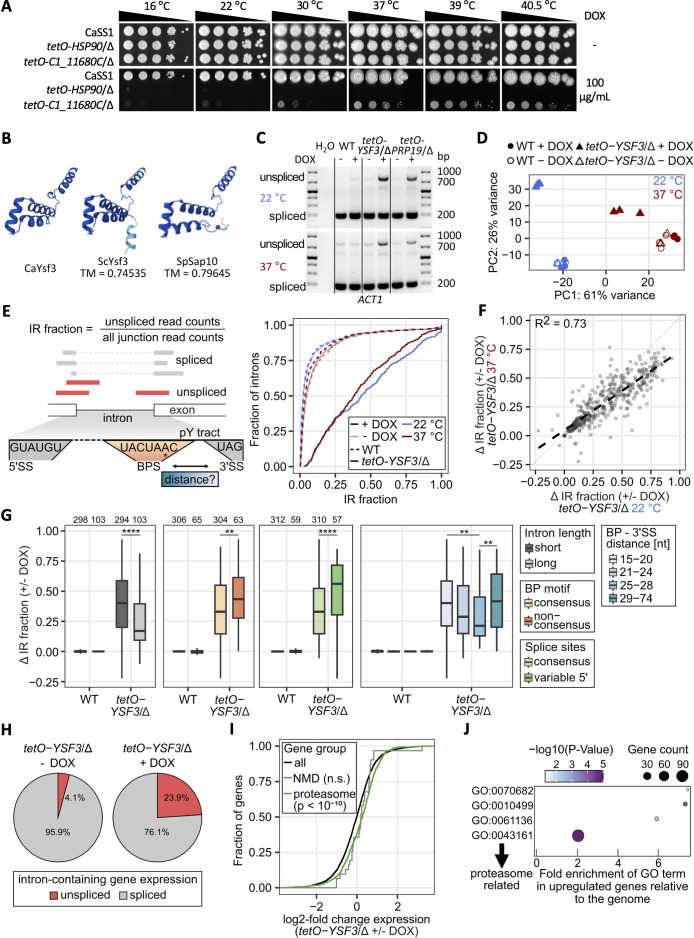
*C1_11680C* encodes Ysf3 in *Candida albicans* and modulates splicing. **A)** Spotting assay performed as described in Fig 2A. **B)** Protein structures of AlphaFold-generated *C. albicans* C1_11680c (UniProt: A0A1D8PF89), *S. cerevisiae* Ysf3 (UniProt: P0C074), and *S. pombe* Sap10 (UniProt: Q9P7R6). Dark blue to yellow to red indicates a reducing confidence score based on the predicted local distance difference test. TM score was calculated by TM-align. A score between 0.5 and 1 implies significant similarity in structure. **C)** Cells were sub-cultured and grown to log phase in the absence or presence of 20 µg/mL DOX at 22 °C or 37 °C before RNA extraction and cDNA synthesis. Primers flanking the *ACT1* intron were used to amplify the spliced and unspliced products from cDNA. **D)** Principal component analysis of gene expression between conditions. The data underlying Fig 3D can be found in S2 Data. **E)** Scheme illustrating intron retention (IR) fraction quantification from RNA-Seq data and intron architecture. The cumulative distribution shows the IR fraction of 389 introns passing the read count cutoff in all samples. The data underlying Fig 3E can be found in S3 Data. **F)** Correlation between Δ IR fraction (+/- DOX) at 37 and 22 °C in *tetO-YSF3*/Δ. The data underlying Fig 3F can be found in S4 Data. **G)** IR difference (± DOX) of wild type (WT) and *tetO-YSF3*/Δ strain at 22 °C of introns categorized by their length, BP motif, splice site motif and BP-to-3′-splice-site-distance, respectively. Top numbers indicate introns per group. BP-3′SS distance groups have equal bin size. Significances obtained using Wilcoxon rank-sum test (intron lengths and motifs) and paired Wilcoxon rank-sum test with Benjamini–Hochberg correction (BP-3′SS distance), ** *p* ≤ 0.01, **** *p* ≤ 0.0001. The data underlying Fig 3G can be found in S4 Data. **H)** Pie chart of unspliced or spliced intron-containing gene expression in *tetO-YSF3*/Δ without and with DOX at 22 °C. The data underlying Fig 3H can be found in S5 Data. **I)** Cumulative distribution of log2-fold change in gene expression between *tetO-YSF3*/Δ with and without DOX for the indicated gene groups at 22 °C. Significances are relative to the ‘all’ genes (Kolmogorov–Smirnoff test). The data underlying Fig 3I can be found in S5 Data. **J)** Significantly enriched ‘Biological process’ GO terms among upregulated genes relative to the genome. GO: 0070682: proteasome regulatory particle assembly; GO: 0010499: proteasomal ubiquitin-independent protein catabolic process; GO: 0061136: regulation of proteasomal protein catabolic process; GO: 0043161: proteasome-mediated ubiquitin-dependent protein catabolic process. The data underlying Fig 3J can be found in S6 Data. Numerical data and source gel images underlying this Figure can be found in S1 Data and S1 File Raw_Images.

While the ability of *C. albicans* to survive within the human host requires it to thrive at physiological and febrile temperatures, this organism also causes a significant number of chronic mucocutaneous infections on the skin, nails, and other mucous membranes, and can survive on different environmental surfaces [40,41]. This requires *C. albicans* to grow at temperatures below 37 °C. Thus, to investigate which genes are specifically important for growth at lower temperature (cluster 3), we focused on the uncharacterized gene *C1_11680C* lacking functional annotation on *CGD* [28]. Using spot dilution assays on YPD agar plates with and without DOX, we confirmed transcriptional repression of *C1_11680C* impaired fitness at all temperatures, with a more severe growth defect observed at temperatures below 37 °C (Figs 3A and S2A). While we were unable to identify a potential homolog based on DNA sequence comparisons with *S. cerevisiae,* BLASTp searches against *S. cerevisiae* and *Schizosaccharomyces pombe* genomes predicted C1_11680c as a splicing factor (Ysf3) belonging to the SF3b subcomplex of the U2 snRNP [42–45]. Template modeling (TM) of the predicted *C. albicans* C1_11680c structure with *S. cerevisiae* Ysf3 and *S. pombe* Sap10 by AlphaFold [46] yielded TM-align [47] scores above 0.7, supporting C1_11680c as Ysf3 in *C. albicans* (Fig 3B).

In *S. cerevisiae*, the SF3b complex consists of six subunits (Hsh155, Cus1, Rse1, Hsh49, Ysf3, and Rsd3) that play a role in recognizing branch point sequences on pre-mRNA during the early stages of spliceosome assembly [48]. Spot dilution assays confirmed that across all temperatures depletion of *RDS3* and *RSE1* had little to no impact on fitness, whereas depletion of *HSH49* and *HSH155* caused severe growth defects, with *HSH155* being essential (S2A and S5A Figs). Interestingly, depletion of *CUS1* resulted in similar growth phenotype across all temperatures tested to depletion of *YSF3* (S5A Fig). This highlights the differential dependency of various splicing genes in fitness across different temperatures.

To test the hypothesis that *C1_11680C* encodes Ysf3 and is important for *C. albicans* splicing*,* we examined the splicing efficiency of the *ACT1* transcript upon depletion of *YSF3*. We also included the *tetO-PRP19*/Δ strain, as *PRP19* is known to be important for splicing of *ACT1* in *C. albicans* [49]. To avoid potential off-target effects by DOX, 20 µg/mL was used, which was sufficient to deplete *YSF3* transcript levels in the *tetO-YSF3*/Δ GRACE strain (S5C Fig) [50]. *ACT1* was efficiently spliced in the wild-type control in the absence or presence of DOX, suggesting DOX has no impact on splicing efficiency of the *ACT1* transcript (Fig 3C). In the *tetO-YSF3*/Δ and *tetO-PRP19*/Δ strains, we observed an accumulation of unspliced *ACT1* in the presence of DOX at both 22 and 37 °C, supporting that Ysf3 is important for splicing in *C. albicans* (Fig 3C).

While it has been previously reported that high temperatures reduce splicing efficiency in *C. albicans* leading to enhanced intron retention (IR) [49], the link between low temperature (temperatures below 30 °C) and splicing efficiency has yet to be investigated. To explore the impact of low temperature on global IR and assess the importance of Ysf3 for global pre-mRNA splicing, we employed 150 bp paired-end RNA-Seq on polyadenylated RNA to map splicing of all intron-containing genes using the *tetO-YSF3*/Δ GRACE strain and the wild-type control in the presence and absence of 20 µg/mL DOX at both 22 and 37 °C. Principal component analysis of gene expression profiles showed that biological replicates behaved similarly and clustered together (Fig 3D). Furthermore, a significant reduction of *YSF3* RNA expression upon DOX treatment led to altered gene expression profiles and the formation of distinct clusters at the two tested temperatures (Fig 3D and S2 Data). Confirming the RT-PCR results (Fig 3C), we detected increased intronic reads for *ACT1* when *YSF3* was depleted by DOX (S6A Fig). To assess the degree of pre-mRNA splicing globally, we performed a junction-read based analysis to estimate the fraction of IR in all 8 conditions (Fig 3E). This revealed that most introns are well spliced in wild type and the *tetO-YSF3*/Δ strain in the absence of DOX (median IR fraction for WT: 0.02 (22 °C)/0.04 (37 °C); for *tetO-YSF3*/Δ without DOX: 0.02 (22 °C)/0.05 (37 °C)) (S3 Data). A modest, but significant increase in IR was seen at 37 °C in wild type and the *tetO-YSF3*/Δ GRACE strain without DOX, as reported previously (Wilcoxon rank-sum test, *p* < 0.0001****, Figs 3E and S6B) [49]. However, depletion of *YSF3* at both temperatures increased IR substantially to median IR fractions of 0.43 and 0.39 at 22 °C and 37 °C, respectively (Fig 3E). IR differences upon depletion of *YSF3* were similar and well correlated between all intron-containing genes at both temperatures; however, slightly more pronounced at 22 °C than at 37 °C (Fig 3F and S4 Data).

According to the function of *S. cerevisiae* Ysf3 within the SF3b complex, we hypothesized that *C. albicans* Ysf3 could be involved in recognizing branchpoint sequence (BPS) and influence splicing of introns with diverse *cis*-regulatory features as the U2 snRNP [45,48]. To assess this, we analyzed the impact of *YSF3* depletion on the accumulation of introns with diverse features, such as intron length, BPS to 3′ splice site (SS) distance, SS consensus, polypyrimidine tract length, and composition and intronic GC-content (S6C and S6D Fig and S4 Data). All features could be extracted from the annotated intronic sequence (Methods). To analyze *C. albicans* BPSs, we scored intronic 7-nt (nucleotide) motifs according to their similarity to the 7-nt BPS found in *S. cerevisiae* and also reported for *C. albicans* [51,52]. For most introns, a single motif close to the intron end (3′ SS) was detected, consistent with the evolutionarily conserved distance of BPS and 3′ SS [52]. For introns with multiple high-scoring 7-nt motifs, the motif was chosen that had the closest match to the typically observed distance of 15−74 nts (median 24 nts) to the 3′ SS and the higher score. As reported previously*,* the consensus BPS is 5′-UACUAAC-3′ and can be found in >80% of introns (S3 Data) [51,52]. Consistent with our hypothesis, IR and IR differences were increased for introns with non-consensus BPSs and for BPSs that are more distal or proximal to the 3′ SS than the typical branchpoint at both temperatures (Figs 3G and S6D). Interestingly, variation in other intronic features, such as intron length, and 5′ SS sequence also correlated with variation in IR difference (Figs 3G and S6C and S6D). This analysis suggests *C. albicans* Ysf3 is an important splicing factor, including for those introns that are short and/or exhibit nonconsensus *cis*-regulatory elements.

Given the high prevalence of unspliced mRNAs upon *YSF3* depletion, the incompletely processed mRNAs could produce incomplete and possibly toxic, truncated or misfolded proteins that could impact growth. Combining mRNA expression and IR estimates for all intron-containing genes passing our minimal read depth cutoffs for splicing and expression quantification, we estimated that the pool of unspliced, adenylated, and possibly translated cytoplasmic mRNAs increased 6-fold (from 4.1% to 23.9% at 22 °C) and 4-fold (from 5.2% to 20.3% at 37 °C) upon *YSF3* depletion (Figs 3H and 6E and S5 Data). This could lead to an increase of prematurely terminated translation products that could be cleared by nonsense-mediated decay (NMD) or require increased proteasome capacity [53,54]. Although we did not see evidence for increased expression of NMD components, genes associated with proteasomal function were increased in their expression relative to all expressed genes and enriched in the group of upregulated genes (Figs 3I, 3J, and S6F and S6 Data). Among downregulated genes, genes involved in filamentous growth and cellular responses to the environment were enriched, consistent with the previously established link between splicing and filamentation [49] (S6G Fig). Interestingly, depletion of *YSF3* significantly reduced filamentation in YPD supplemented with 10% newborn calf serum at 37 °C compared to wild-type controls (S5B and S5C Fig). This phenotype was similar to what was observed upon depletion of *EFG1,* a transcription factor known to be required for filamentation. Overall, we conclude that CaYsf3 is important for splicing of most intron-containing genes with particular importance for short introns and/or nonconsensus *cis*-regulatory elements.

### Rapid evolution suggests *C6_00110C* plays a key role in genome stability in *C. albicans*

**Fig 4:**
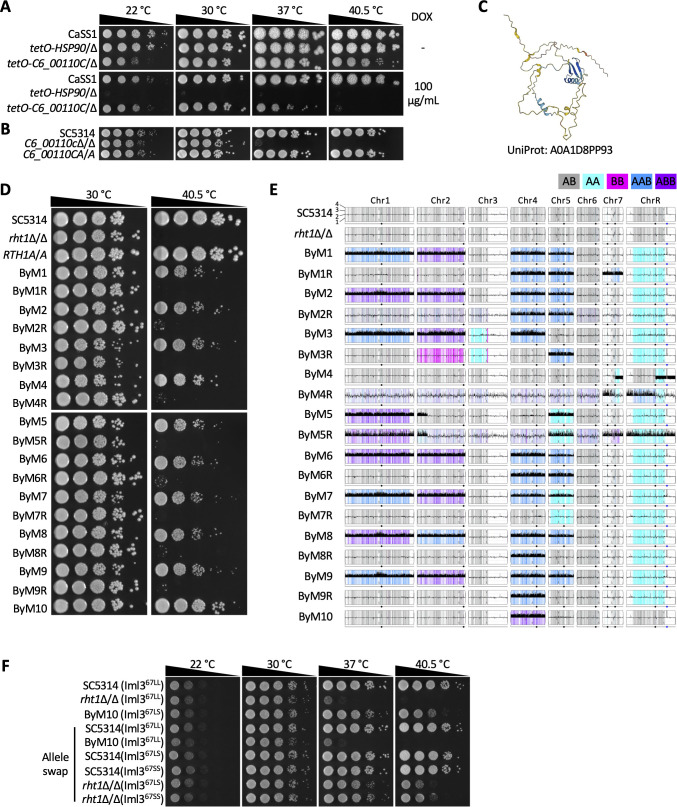
The importance of *C6_00110C* for high temperature growth can be bypassed via rapid evolution. **A)** Spotting assay was performed as described in Fig 2A except plates were incubated for 2 days. **B)** Spotting assay was performed as described in Fig 2A. *C6_00110CA*/*A* represents the *C6_00110c*Δ/Δ strain which was complemented with both A alleles. **C)** Protein structure of AlphaFold-generated *Candida albicans* C6_00110c (UniProt: A0A1D8PP93). The color scale from dark blue to yellow to red indicates a reducing confidence score based on the predicted local distance difference test (pLDDT). **D)** Spotting assay was performed as described in panel A. **E)** Y-MAP plot visualizing chromosome copy number and loss of heterozygosity events from WGS analysis. Haplotypes relative to the reference genome SC5314 are color-coded (see legend). **F)** Spotting assay was performed as described in panel A except plates were imaged after 24 h.

Uncharacterized *Candida* clade-specific genes essential for growth can enable discovery of new biology relevant to developing antifungal treatments. Hence, we turned to a *Candida* clade-specific gene *C6_00110C* that was identified as important for growth at elevated temperature (cluster 6). As observed by spot dilution assays, transcriptional repression of *C6_00110C* with DOX abrogated growth only at 40.5 °C (Fig 4A). To confirm the temperature-specific essentiality phenotype, we generated a homozygous deletion mutant of *C6_00110C,* as well as a complemented strain. When examining the growth phenotype of these mutants, we confirmed that deletion of *C6_00110C* resulted in little to no growth at temperatures at or above 37 °C (Fig 4B). Thus, we named the gene Required for Heat Tolerance 1 (*RHT1*). BLASTp analysis, conserved domain searches, and co-expression data [55] failed to predict a function for Rht1 and AlphaFold prediction yielded a structure with low model confidence scores for the majority of the amino acids in the protein sequence (pLDDT < 70) (Fig 4C), leaving the function of this *Candida-*specific protein elusive. Thus, we turned to our bypass mutant selection strategy to uncover the function of this elusive gene and plated 10^8^ *rht1*Δ/Δ cells on YPD agar plates and incubated at 42 °C for 5 days. Through this approach, we evolved 10 independent bypass mutants (ByMs) with a restored ability to grow at 42 °C in the absence of *RHT1* (Fig 4D). Interestingly, when these ByMs were passaged at 30 °C, colonies with restored high-temperature sensitivity (ByMRs) were readily isolated (S4B Fig). ByMRs were successfully isolated for nine out of the 10 ByMs (Figs 4D and S4B).

To decipher the genetic mechanisms governing the growth phenotypes observed for both the ByMs and ByMRs, we performed whole genome sequencing of all relevant lineages. When examining the genomic changes of the ByMs, we observed significant chromosomal copy number changes for all ByMs. In particular, we detected trisomy of Chr1, Chr2, Chr4 and Chr5 for ByMs 1, 2, 6, 7, 8, and 9; trisomy of Chr1, Chr2, and Chr4 for ByM3; segmental monosomy of Chr7 and ChrR for ByM4; and trisomy of Chr1 and Chr5 as well as segmental trisomy of Chr2 for ByM5 (Fig 4E). When analyzing chromosome copy numbers for ByMRs including 1R, 2R, 3R, 6R, 7R, 8R, and 9R, all strains shared the concurrent loss of Chr1 and Chr2 trisomies, despite differences in Chr3, Chr4, and Chr7 trisomies (Fig 4E). ByM4R and ByM5R gained segmental and full length Chr7 and ChrR trisomies, respectively (Fig 4E). While aneuploidies were detected for ByM10, variant-calling identified a heterozygous mutation in *IML3*. Iml3 was recently characterized as a *C. albicans* kinetochore component and is required for heat tolerance [27]. The identified mutation resulted in a substitution of leucine (L) to serine (S) at the amino acid position 67 (Fig 4F). Substitution of S back to L at position 67 in ByM10 abrogated growth at high temperature, whereas both heterozygous and homozygous substitution of L to S at position 67 in the *rht1*Δ/Δ background restored growth at 40.5 °C, confirming the mutation in Iml3 was both necessary and sufficient for bypassing high-temperature essentiality in *rht1*Δ/Δ (Fig 4F). Overall, the ploidy changes and mutation in a kinetochore gene observed in *rht1*Δ/Δ mutants grown at elevated temperature suggests Rht1 may be involved in chromosome segregation and/or genome stability.

**Fig 5:**
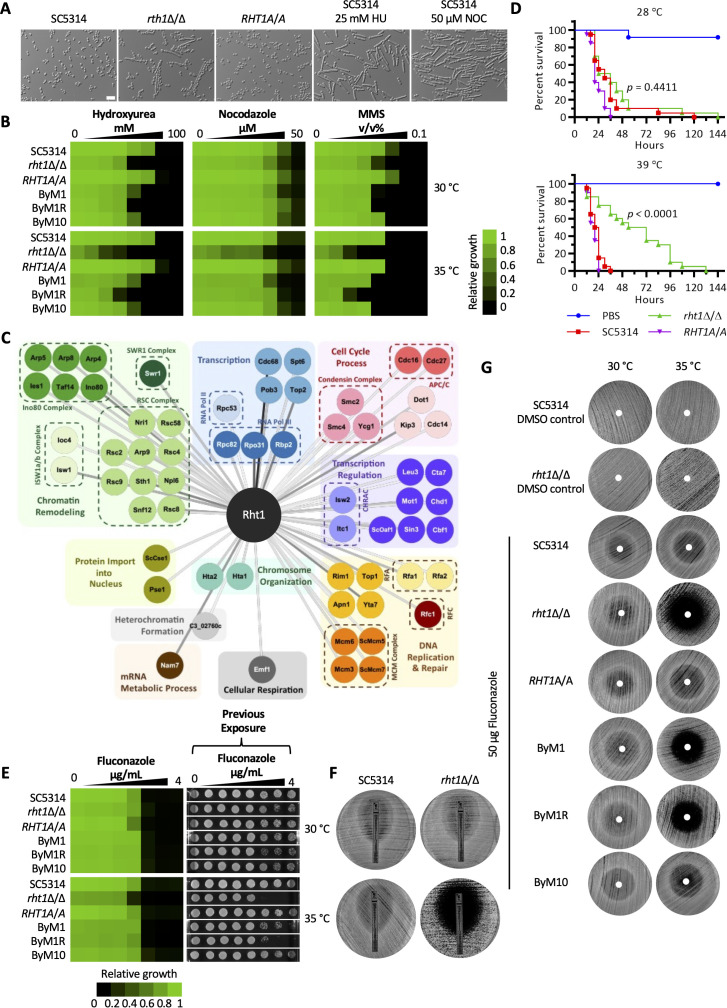
Rht1 functions in cell cycle progression and modulates fluconazole susceptibility. **A)** *Candida albicans* strains were sub-cultured to an OD_600_ of 0.2 in YPD and grown at 30 °C for 4 h. Cells treated with 25 mM hydroxyurea (HU) or 50 µM nocodazole (NOC) were used as controls. Scale bar represents 20 µm. **B)** Dose-response assays were performed with 2-fold titrations of hydroxyurea, nocodazole, and methyl methanesulfonate (MMS) at 30 and 35 °C. OD_600_ was measured after 48 h, values were normalized relative to wild type without drug (see color scale bar). **C)** Significant interactions for Rht1-GFP were defined through SAINTexpress analysis compared with the Eno1-GFP control. (BFDR threshold = 0, average spectral counts ≥ 20, fold change relative to Eno1 ≥ 50). Protein-labeled nodes are colored based on protein function annotations. Line color ranging from dark black to light gray reflects the decreasing fold change in peptide count of Rht1 relative to Eno1. The data underlying Fig 5C can be found in S7 Data. **D)** *G. mellonella* (20 larvae per group) were inoculated with 1 × 10^6^ *C. albicans* cells. Larvae were incubated at 28 and 39 °C and monitored for survival. Log rank (Mantel-Cox) test assessed significant difference between wild type and *rht1*Δ/Δ. **E)** Dose-response assay was performed as in B. After 48 h, 5 μL was removed from liquid cultures and spotted onto drug-free agar to assess cidality. Plates were placed in 30 °C for 48 h. **F)** Wild-type (SC5314) or *rht1*Δ/Δ cells were spread on YPD agar and an ETEST strip of fluconazole was applied. Plates were incubated at 30 or 35 °C and imaged after 3 days. **G)** Strains were spread on YPD agar and 50 µg of fluconazole was spotted on filter paper. Plates were incubated at 30 or 35 °C and imaged after 3 days. The data underlying Fig 5B, 5D, and 5E can be found in S1 Data.

Given a potential role for Rht1 in genome stability, we examined whether this gene was important for cell cycle progression. Interestingly, the *rht1*Δ/Δ strain produced both yeast and filaments under non-filament-inducing conditions with the filaments resembling wild-type cells treated with cell cycle inhibitors hydroxyurea (HU) or nocodazole (NOC), suggesting a defect in cell cycle progression in the absence of *RHT1* (Fig 5A). Furthermore in a dose-response assay, deletion of *RHT1* resulted in a higher sensitivity to G1/S phase inhibitors HU and methyl methanesulfonate (MMS) [56,57] and the G2/M phase inhibitor NOC [58], supporting the potential role of Rht1 in cell cycle progression (Fig 5B). Higher temperature further exacerbated sensitivity of the *rht1*Δ/Δ mutant to all three drugs (Fig 5B), reinforcing the greater requirement of cell cycle-related genes at high temperature. While the complemented strain fully restored tolerance to all three drugs, ByM1 and ByM10 only partially restored tolerance to HU and MMS (Fig 5B).

Identification of protein interaction partners can provide further insights into gene function. Hence, we performed affinity purification coupled with mass spectrometry (AP-MS) of a C-terminally GFP-tagged Rht1 strain to define the protein’s interactome using Eno1-GFP as a control for background [25,27]. The homozygous tagged protein was expressed and fully rescued the *rht1*Δ/Δ high-temperature essentiality phenotype, confirming functionality of Rht1-GFP (S7A and S7B Fig). AP-MS identified 62 high-confidence protein interactors (Bayesian False Discovery Rate [BFDR] = 0, average spectral counts ≥20, and fold change relative to Eno1 ≥50; S7 Data). Consistent with our genetic evidence, the majority of the interactors were found to function in chromatin remodeling, DNA replication and repair, cell cycle progression, transcription, and transcription regulation (Fig 5C). A total of 29 genes encoding Rht1-interacting proteins were included in the secondary fitness screen across six different temperatures. Twelve genes were found in cluster 1 (genes important for fitness across all temperatures) and 15 genes were found in clusters 2 or 6 (genes important for fitness at elevated temperatures), including six genes encoding proteins of the Remodels the Structure of Chromatin (RSC) complex (S7C Fig). Interestingly, five of the six RSC complex members are located on Chr1 or Chr2, chromosomes that increased in copy number upon growth of the *rht1* mutant at elevated temperature.

### Rht1 affects *C. albicans* virulence and influences fluconazole activity

The specificity of Rht1 to the *Candida* clade, its requirement for growth at high temperatures, and its implication in genome stability motivated assessment of its role in virulence and antifungal susceptibility. To determine the role of Rht1 in *C. albicans* virulence, we turned to the *G. mellonella* model of *C. albicans* infection. While injection of the wax moths with the wild-type and complemented strains resulted in profound lethality, the *rht1*Δ/Δ strain had significantly attenuated virulence only at 39 °C, confirming that Rht1 is required for *C. albicans* pathogenesis at febrile temperature (Fig 5D).

Genome stability modulates fluconazole susceptibility in *C. albicans* [59], and therefore, we wanted to investigate whether Rht1 was important for azole activity. Dose-response assays with fluconazole showed that loss of Rht1 rendered fluconazole cidal only at 35 °C (Fig 5E). Similarly, when we performed an ETEST strip assay and disc diffusion assay by spotting 50 µg of fluconazole onto a disc placed on a lawn of *C. albicans,* we confirmed that Rht1 is important for azole activity at 35 °C, as the deletion mutant grew in the absence of fluconazole at 35 °C and showed enhanced clearance around the fluconazole strip or disc relative to wild type (Fig 5F and 5G). The zone of clearance remained after 5 days of incubation at 35 °C (S8A and S8B Fig). Interestingly, growth around the fluconazole disc was restored in ByM10 at 35 °C but not in ByM1 (Fig 5G), suggesting aneuploidy could not fully mask the requirement of Rht1 in fluconazole susceptibility, but the substitution in Iml3 could.

## Discussion

Since its original release in 2003, two subsequent expansions of the GRACE library, including the set of 1,235 genes introduced in this study, have increased the total coverage to 4,492 genes [23,25], representing the largest mutant library in *C. albicans*. Screens of subsets of the GRACE library have been instrumental in advancing our understanding of *C. albicans* biology and pathogenesis mechanisms including gene essentiality [23,25], fitness in different environmental conditions [27], morphogenesis [22,24,49,60,61], adhesion [62], macrophage interactions [63], and antifungal susceptibility [64,65]. With our expanded GRACE collection, we sought to identify genes required for fitness in diverse temperatures in *C. albicans* by screening the collection of mutants at both low and high temperatures. Our functional genomic screen identified dozens of genes with important roles in governing *C. albicans* fitness in a temperature-dependent manner, including *GAR1*, *YSF3*, and *RHT1.*

Gar1 plays an important function in pseudouridylation in *S. cerevisiae*. In *C. albicans*, deletion of *GAR1* caused a severe growth defect across all temperatures tested and completely abrogated growth at 40.5 °C, indicating that *GAR1* is important for fungal survival. Interestingly, the nonsynonymous mutation in *KRR1* was able to compensate for the fitness defect of the *gar1*Δ/Δ strain. Krr1 functions in protein and RNA binding via two KH domains for ribosome assembly [66]. The nonsynonymous mutation in the evolved mutant is located in the first KH domain, which lacks the typical GXXG RNA-binding motif [67]. The mutation could either directly or indirectly prime Cbf5 for its catalytic activity in the absence of Gar1. Supporting this hypothesis, Krr1 was shown to physically interact with Cbf5 in multiple studies in *S. cerevisiae* [38,66,68–70]. In *S. cerevisiae*, a point mutation in the conserved XLD motif of *CBF5* abolishes its pseudouridine synthase activity and eliminates rRNA pseudouridylation, but the mutant remains viable [71]. Thus, whether Gar1 and Krr1 impact temperature-dependent growth via rRNA pseudouridylation in *C. albicans* remains to be elucidated.

Initially thought to be an obligate human commensal fungal pathogen, *C. albicans* has been found to persist in the environment [72–74], expanding its thermal adaptation range. In this study, we showed that genes in cluster 3 are enriched in RNA metabolic processes and rRNA processes, and they are important for tolerance to temperatures lower than the host temperature of 37 °C. *C1_11680C* grouped with cluster 3 in the secondary screen, and spot dilution assays confirmed that *C1_11680C* depletion resulted in no growth at 16 °C and severe growth defects at all other temperatures. Functional characterization of *C1_11680C* revealed that the gene likely encodes Ysf3, a component of the SF3b complex required for splicing. RNA-sequencing analyses at 22 and 37 °C showed that depletion of *YSF3* reduced the efficiency of pre-mRNA splicing, likely increasing the translation of truncated proteins. The effect was slightly stronger at the lower temperature, correlating with a more pronounced growth phenotype at this condition. The high protein structure similarity of *C. albicans* Ysf3 to *S. cerevisiae* Ysf3 coupled with the reduced splicing efficiency upon *YSF3* depletion suggests that Ysf3 has an important role for splicing in *C. albicans*. However, future studies are needed to experimentally confirm its function as part of the Sf3b complex and its possible involvement in BP recognition.

Among the 30.6% of genes without functional annotation in the GRACEv3 collection is *C6_00110C* (*RHT1*), which we identified as a gene required for *C. albicans* fitness at high temperature. Compared with the *gar1*Δ/Δ strain, the *rht1*Δ/Δ strain exhibited an astonishing capacity to evolve bypass mutants, overcoming the fitness defect of the strain at elevated temperatures. Nine out of the 10 bypass mutants were driven by aneuploidy, and seven shared Chr1 and Chr2 trisomies. Interestingly, all seven bypass mutants lost Chr1 and Chr2 trisomies when passaged at a lower temperature, suggesting Chr1 and Chr2 trisomy may contribute to the bypass mechanism. The dynamic ploidy changes observed at different temperatures in *rht1*Δ/Δ mutants suggest that Rht1 may play a role in maintaining genome stability; however, the temperature-dependent effects of Rht1 on genome stability remain to be elucidated. Among the 62 high-confidence Rht1 interactors, 10 proteins function in the RSC chromatin remodeling complex and 8 are encoded by genes located on Chr1 and Chr2 [75]. It is possible that in *rht1*Δ/Δ mutants, upregulation of RSC complex genes on Chr1 and Chr2 could enhance chromatin remodeling activities. Supporting this hypothesis, experimental evolution for high temperature survival identified mutations in the chromatin remodeling SWI/SNF complex that enables thermal adaptation in *S. cerevisiae* [76]. However, additional experiments are warranted to confirm this model.

The evolvability of bypass mutants in the *rht1* background highlights that *C. albicans* is able to employ diverse mechanisms in mitigating environmentally contingent detrimental mutations. Such phenomena have also been described in *C. albicans* when examining the ability of this species to evolve resistance to the azoles [59]. Aneuploidy formation, in particular, isochromosome formation on the left arm of chromosome 5, drives fluconazole resistance [59,77,78]. Chromosome 5 contains *ERG11* and *TAC1*, encoding the fluconazole target and a transcription factor regulating drug efflux pumps [79]. Interestingly, the aneuploidy identified in ByM1 failed to restore azole susceptibility despite the increased copy number of Chr5. In contrast, the evolved mutation in *IML3* identified in ByM10 largely restored fungal viability in the presence of fluconazole, suggesting Rht1 likely exerts pleiotropic functions that regulate fitness at elevated temperature and azole exposure.

Identification of Rht1 as a novel *Candida* clade-specific gene required for heat tolerance and virulence highlights the power of functional genomic screens to advance our understanding of *C. albicans* biology and the importance of continuous expansion efforts to reach whole-genome coverage. Despite our functional characterizations of Rht1, the exact molecular mechanism underlying its role in cell cycle progression and genome stability remains elusive. Interestingly, the functionally relevant kinetochore protein Iml3, implicated by bypass mutant selection, was not present in the Rht1 interactome elucidated by AP-MS. Similarly, close examination of the Iml3 interactome failed to identify Rht1 [27], suggesting a lack of physical interaction between the two proteins. Additional experiments are warranted to pinpoint Rht1’s functional link with the *C. albicans* kinetochore.

Overall, our work characterized genes required for fitness in diverse temperature environments for the major fungal pathogen *C. albicans*. While connections between temperature and antifungal resistance have been described in the literature [80], more examples continue to be unveiled. In the recently discovered fungal pathogen *Rhodsporidiobolus fluvialis*, human body temperature elevates mutation rates and drives pan-drug resistance against all three classes of antifungal drugs [81,82]. In *Cryptococcus deneoformans*, heat stress enhances transposon mobility, which may enable adaptation during infection [83,84]. Thus, it is crucial to continue to probe the mechanisms by which thermal tolerance enables antifungal resistance to disarm fungal threats.

## Materials and methods

### Fungal strains and growth conditions

Strains used in this study are listed in S1 Table. Plasmids used in this study are listed in S2 Table. Strains were frozen in 25% glycerol at −80 °C for long-term storage. Active cultures were maintained on solid yeast extract peptone (YPD; 1% yeast extract, 2% bactopeptone, 2% glucose, and 2% agar) at 4 °C for no longer than 1 month. Liquid cultures were grown in YPD at 30 °C, unless otherwise indicated. To select for NAT-resistant mutants, NAT (Jena Bioscience, 96736-11-7) was solubilized in water and supplemented into YPD agar plates (150–250 μg/mL). Prototrophic colonies were selected on SD plates (0.674% yeast nitrogen base without amino acids with ammonium sulfate, 2% glucose, and 2% agar). Temperature-dependent fitness screens were performed using minimal medium plates (0.17% yeast nitrogen base without amino acids and without ammonium sulfate, with 2% glucose, 0.1% glutamic acid (MSG), 0.002% histidine, and 2% agar). DOX (Bio Basic, DB0889) stock was prepared at a concentration of 50 mg/mL in H_2_O, filter-sterilized, and added to the media at the indicated concentration.

### Manual curation of gene ontology (GO) annotation by evidence code

All genes in the *C. albicans* genome were analyzed for GO annotations by biological functions on *CGD* website [28]. Genes with GO terms assigned with codes IC (inferred by curator), IDA (inferred by direct assay), IEP (inferred from expression pattern), IGI (inferred from genetic interaction) in *C. albicans,* IMP (inferred from mutant phenotype), IPI (inferred from physical interaction), and TAS (traceable author statement) were categorized in the “experimentally characterized” group. Genes with GO terms assigned based on evidence in *S. cerevisiae* with codes IEA (inferred from electronic annotation), IGI, ISA (inferred from sequence alignment), ISM (inferred from sequence model), ISO (inferred from sequence orthology), and ISS (inferred from sequence or structural similarity) were categorized in the “putative based on *S. cerevisiae* ortholog” group. Genes with GO terms assigned based on evidence in *S. pombe* with code IEA were categorized in the ‘putative based on *S. pombe* ortholog’ group. Genes with GO terms assigned based on evidence in other fungal species with codes IEA, ISA, ISO, and ISS were categorized in the ‘putative based on other fungal species’ group. Genes with GO terms assigned based on conserved protein domains with codes IEA were categorized in the “putative based on conserved protein domains” group. Genes with GO terms assigned codes ISA based on nonfungal species, ISM, and NAS (nontraceable author statement) were categorized in the ‘other’ group. Genes with no GO terms assigned were categorized in the “no data available” group.

### Strain construction

#### Heterozygous (HET) deletion mutant library expansion.

Double-barcoded HET mutants that were not present in the original collection were constructed, as described previously [25]. Briefly, the *CaHIS3* cassette was PCR amplified from pLC1251 using primer 5 and primer 6, each containing 43 base pairs of homology to the 5′ or 3′ of the target gene, internal unique 20 base pair strain-identifying barcodes with flanking common sequences, and 18 base pairs that anneal to the 5′ or 3′ of the *CaHIS3* cassette. The cassette was transformed into the histidine auxotrophic parental strain, CaLC6106, that contains the tetracycline-repressible transactivator. Prototrophic colonies were PCR tested using the primer 3 and oLC6701 and primer 7 and oLC8017 for upstream and downstream integration, respectively. Primer sequences used in this study are listed in S8 Data.

#### GRACE library expansion.

GRACE strains were generated from HET mutants, as described previously [25]. Briefly, the *tetO* cassette containing the NAT marker was PCR amplified from pLC763 using primer 1 and primer 2, each containing 20 base pairs complementary to the *tetO* cassette and 70 base pairs of homology immediately upstream or downstream of the start codon of the target gene. The *tetO* cassette was transformed into the corresponding HET mutant. NAT-resistant colonies were PCR tested for disruption of the wild-type promoter with the *tetO* cassette using primer 3 and primer 4 that anneals ~250–350 base pairs upstream and downstream of the start codon, respectively. Downstream integration of the *tetO* promoter was confirmed by PCR using primer oLC2535 that anneals within the *tetO* cassette and primer 4.

#### CaLC10712.

*GAR1* coding sequence (CDS) was deleted using a transient CRISPR approach adapted from Min and colleagues [85]. A flippable *SAT1* cassette [86] was PCR amplified from XmnI linearized pLC49 using oLC11772 and oLC11773 containing 80 base pairs of homology to the upstream or downstream of *GAR1* CDS and 20 base pairs annealing to the *FLP-SAT1* cassette. The *CaCAS9* cassette was amplified from pLC1081 using oLC6924 and oLC6925. The sgRNA fusion cassette was PCR amplified from pLC1081 with oLC6926 and oLC11770 (fragment A) and oLC6927 and oLC11771 (fragment B), and fusion PCR was performed on fragments A and B using the nested primers oLC6928 and oLC6929. The *FLP-SAT1* cassette, sgRNA, and Cas9 DNA were transformed into CaLC155 (SC5314) to generate NAT resistant *GAR1* deletion mutant. Upstream integration was PCR tested using oLC275 and oLC11774, and downstream integration was tested using oLC274 and oLC11775. Lack of a wild-type allele was PCR tested using oLC11776 and oLC11777. The NAT-sensitive CaLC10712 was derived from the above mutant by *SAT1* cassette excision mediated by the site-specific recombinase FLP. *SAT1* excision was confirmed by PCR using oLC11774 and oLC11775.

#### CaLC10713.

*GAR1* CDS was cloned into pLC2052 using the Gibson assembly method [87]. In brief, plasmid backbone was PCR amplified with oLC12200 and oLC12206 from pLC1330 [88], *GAR1* CDS and 3′UTR sequence were PCR amplified from SC5314 genomic DNA with oLC12201 and oLC12202 and oLC12205 and oLC12207, respectively, *SAT1* cassette was PCR amplified from pLC49 with oLC12203 and oLC12204, and DNA fragments with 20–40 base pairs of overlapping sequences were assembled together using the HiFi assembly kit (NEB, E5520S). The sequence of pLC2052 was confirmed by Sanger sequencing. The *GAR1-SAT1* cassette was PCR amplified from pLC2052 with oLC12201 and oLC12207. The *CaCAS9* cassette was amplified from pLC1081 using oLC6924 and oLC6925. The sgRNA fusion cassette was PCR amplified from pLC1081 with oLC6926 and oLC11150 (fragment A) and oLC6927 and oLC11151 (fragment B), and fusion PCR was performed on fragments A and B using the nested primers oLC6928 and oLC6929. The *GAR1-SAT1* cassette, sgRNA, and Cas9 DNA were transformed into CaLC10712. Upstream integration was PCR tested using oLC275 and oLC12311, and downstream integration was tested using oLC274 and oLC12312. Lack of the *gar1*Δ::*FRT* allele was PCR tested using oLC11954 and oLC11955.

#### CaLC10720.

*RHT1* CDS was deleted using a transient CRISPR approach adapted from Min and colleagues [85]. A flippable *SAT1* cassette [86] was PCR amplified from XmnI linearized pLC49 using oLC12363 and oLC12361 containing 80 base pairs of homology to the upstream or downstream of *RHT1* CDS and 20 base pairs annealing to the *FLP-SAT1* cassette. The *CaCAS9* cassette was amplified from pLC1081 using oLC6924 and oLC6925. The sgRNA fusion cassette was PCR amplified from pLC1081 with oLC6926 and oLC12362 (fragment A) and oLC6927 and oLC12363 (fragment B), and fusion PCR was performed on fragments A and B using the nested primers oLC6928 and oLC6929. The *FLP-SAT1* cassette, sgRNA, and Cas9 DNA were transformed into CaLC155 (SC5314) to generate NAT resistant *RHT1* deletion mutant. Upstream integration was PCR tested using oLC275 and oLC12436, and downstream integration was tested using oLC274 and oLC12437. Lack of a wild-type allele was PCR tested using oLC12364 and oLC12365. The NAT-sensitive CaLC10720 was derived from the above mutant by *SAT1* cassette excision mediated by the site-specific recombinase FLP. *SAT1* excision was confirmed by PCR using oLC12436 and oLC12437.

#### CaLC10721 and CaLC10748.

*RHT1* CDS and *RHT1-GFP* cassette were transformed into the *rht1*Δ::*FRT*/*rht1*Δ::*FRT* and SC5314 strains to generate CaLC10721 and CaLC10748 using a transient CRISPR approach adapted from Min and colleagues [85]. To generate the *RHT1-SAT1* cassette, *RHT1* CDS was PCR amplified from SC5314 genomic DNA with oLC12436 and oLC12444, *SAT1* cassette was PCR amplified from pLC1204 with oLC12445 and oLC12439, and fusion PCR was performed on the two PCR products using nested primers oLC12443 and oLC12465. The *RHT1-GFP-SAT1* cassette was PCR amplified from pLC1204 with oLC12438 and oLC12439. The *CaCAS9* cassette was amplified from pLC1081 using oLC6924 and oLC6925. The sgRNA fusion cassette targeting the *RHT1* 3′UTR was PCR amplified from pLC1081 with oLC6926 and oLC12440 (fragment A) and oLC6927 and oLC12441 (fragment B), and fusion PCR was performed on fragments A and B using the nested primers oLC6928 and oLC6929. The *RHT1-SAT1* cassette or the *RHT1-GFP-SAT1* cassette, with sgRNA and Cas9 DNA were transformed into CaLC10720 or SC5314 to generate CaLC10721 or CaLC10748. Upstream integration and downstream integration for CaLC10721 were PCR tested using oLC12436 and oLC6688 and oLC3821 and oLC12442, respectively, and lack of the *rht1*Δ::*FRT* allele was PCR tested using oLC12436 and oLC12442. Upstream integration and downstream integration for CaLC10748 were PCR tested using oLC12446 and oLC10761 and oLC3821 and oLC12442, respectively, and lack of the *RHT1 WT 3'UTR* allele was PCR tested using oLC12446 and oLC12442.

#### CaLC10715 to CaLC10718.

*KRR1*^*109G*^ wild-type CDS (encoding Krr1^37E^) and *KRR1*^*109A*^ mutant CDS (encoding Krr1^37K^) were cloned into pLC2053 and pLC2054 using the Gibson assembly method [87]. In brief, plasmid backbone was PCR amplified with oLC13162 and oLC13163 from pLC1330 [88], *KRR1* CDS was PCR amplified from SC5314 or *gar1*Δ/Δ ByM1 genomic DNA with oLC13164 and oLC13165, *SAT1* cassette was PCR amplified from pLC1204 with oLC13166 and oLC13167, and DNA fragments with 20–40 base pairs of overlapping sequences were assembled together using the HiFi assembly kit (NEB, E5520S). Plasmid sequences were confirmed by sequencing via Plasmidsaurus. *KRR1*^*109G*^*-SAT1* and *KRR1*^*109A*^*-SAT1* cassettes were PCR amplified from pLC2053 and pLC2054 with oLC13164 and oLC13167. The *CaCAS9* cassette was amplified from pLC1081 using oLC6924 and oLC6925. The sgRNA fusion cassette targeting *KRR1* 3′UTR was PCR amplified from pLC1081 with oLC6926 and oLC13160 (fragment A) and oLC6927 and oLC13161 (fragment B), and fusion PCR was performed on fragments A and B using the nested primers oLC6928 and oLC6929. The *KRR1*^*109G*^*-SAT1* cassette, sgRNA, and Cas9 DNA were transformed into SC5314 and *gar1*Δ/Δ ByM1. The *KRR1*^*109A*^*-SAT1* cassette, sgRNA, and Cas9 DNA were transformed into SC5314 and *gar1*Δ/Δ. Lack of wild-type *KRR1* 3′UTR allele was PCR tested using oLC13240 and oLC13241. *KRR1* alleles in CaLC10715 (SC5314 *KRR1*^*109GG*^), CaLC10716 (*gar1*Δ/Δ ByM1 *KRR1*^*109GG*^), CaLC10717 (SC5314 *KRR1*^*109AA*^), and CaLC10718 (*gar1*Δ/Δ *KRR1*^*109AA*^) were confirmed by Sanger sequencing.

#### CaLC10742 to CaLC10747.

*IML3*^*200T*^ wild-type CDS (encoding Iml3^67L^) and *IML3*^*200C*^ mutant CDS (encoding Iml3^67S^) were cloned into pLC2055 and pLC2056 using the Gibson assembly method [87]. In brief, plasmid backbone was PCR amplified with oLC13171 and oLC13172 from pLC1330 [88], *IML3* CDS was PCR amplified from SC5314 or *rht1*Δ/Δ ByM10 genomic DNA with oLC13173 and oLC13174, *SAT1* cassette was PCR amplified from pLC1204 with oLC13175 and oLC13176, and DNA fragments with 20–40 base pairs of overlapping sequences were assembled together using the HiFi assembly kit (NEB, E5520S). Plasmid sequences were confirmed by sequencing with Plasmidsaurus. *IML3*^*200T*^*-SAT1* and *IML3*^*200C*^*-SAT1* cassettes were PCR amplified from pLC2055 and pLC2056 with oLC13173 and oLC13176. The *CaCAS9* cassette was amplified from pLC1081 using oLC6924 and oLC6925. The sgRNA fusion cassette targeting *IML3* 3′UTR was PCR amplified from pLC1081 with oLC6926 and oLC10661 (fragment A) and oLC6927 and oLC10660 (fragment B), and fusion PCR was performed on fragments A and B using the nested primers oLC6928 and oLC6929. The *IML3*^*200T*^*-SAT1* cassette, sgRNA, and Cas9 DNA were transformed into SC5314 and *rht1*Δ/Δ ByM1. The *IML3*^*200C*^*-SAT1* cassette, sgRNA, and Cas9 DNA were transformed into SC5314 and *rht1*Δ/Δ. Lack of wild-type *IML3* 3′UTR allele was PCR tested using oLC10665 and oLC10666. *IML3* alleles in CaLC10742 (SC5314 *IML3*^*200TT*^), CaLC10743 (*rht1*Δ/Δ ByM10 *IML3*^*200TT*^), CaLC10744 (SC5314 *IML3*^*200TC*^), CaLC10745 (SC5314 *IML3*^*200CC*^), CaLC10746 (*rht1*Δ/Δ *IML3*^*200TC*^), and CaLC10747 (*rht1*Δ/Δ *IML3*^*200CC*^) were confirmed by Sanger sequencing.

### Screening GRACE collection for growth at 30 and 39 °C

*C. albicans* GRACE strains in the GRACEv3 library were screened for growth at 30 °C, as previously described [25]. Briefly, GRACE strains were pinned into 96-well plates (Sarstedt) containing liquid YPD medium and grown overnight at 30 °C. The next day, cells were transferred into liquid YPD medium in the absence and presence of 100 µg/mL DOX in technical duplicates and grown at 30 °C overnight. Approximately 2 μL of cultures were then transferred onto minimal media agar plates in the absence and presence of 100 µg/mL DOX using a metal replicator and grown for 2 days 30 °C. The plates were imaged on a ChemiDoc, and growth fitness was scored independently by two people, using the scoring system outlined in Fig 1C. The entire GRACE library was screened for growth fitness at 39 °C following the same screening pipeline except that cells were grown on minimal media agar plates in the absence and presence of 100 µg/mL DOX at 39 °C (S1B Fig).

### Screening prioritized GRACE strains for growth fitness at six temperatures

*C. albicans* GRACE strains with a fitness score above 1 in the presence of DOX from the growth screens at 30 and 39 °C were prioritized for a secondary screen at six temperatures (S1D Fig). 947 strains were arrayed and pinned into 96-well plates (Sarstedt) containing liquid minimal medium in the absence and presence of 0.5 µg/mL DOX and grown overnight at 30 °C. The next day, 5 µL of mixed cells were diluted in 95 µL H_2_O and then 5 µL diluted cells were inoculated onto minimal media agar plates in the absence and presence of 100 µg/mL DOX using a multi-channel pipettor and grown for 10 days at 16 °C, 4 days at 22 °C, 2 days at 30 °C, 2 days at 37 °C, 3 days at 39 °C, and 4 days at 40.5 °C. The plates were imaged on a ChemiDoc, and growth was scored independently by three people, using the scoring system outlined in Fig 1C. Four biological replicates were performed for growth screens at 16 and 22 °C, and three biological replicates were performed for growth screens at 30, 37, 39, and 40.5 °C.

### Clustering and GO enrichment analysis

Genes represented by the 947 strains screened at the six distinct temperatures were clustered based on fitness scores in six temperatures by Euclidean distance (*k* = 6). GO enrichment analysis was performed using CGD for genes in each cluster against the background of 947 genes screened. *P*-value was adjusted by false discovery rate (FDR), and only terms with *p*-adjusted values greater than 0.05 were plotted. Count represents the total number of genes assigned to a given GO term.

### BLAST analysis and protein structure analyses

Blast analyses were performed for *C1_11680C* and *C6_00110C* DNA and protein sequences using NCBI (https://blast.ncbi.nlm.nih.gov/Blast.cgi) [89], FungiDB (https://fungidb.org/fungidb/app/workspace/blast/new) [90], and SGD (https://www.yeastgenome.org/blast-sgd) [91] blast tools. AlphaFold [46] predicted structures were obtained from UniProt31 Website (https://www.uniprot.org/). Similarity between pairs of protein structures was assessed through TM-Align (https://zhanggroup.org/TM-align/) [47].

### Spot dilution assay

Wild-type and GRACE strains were grown in YPD in the absence and presence of 0.05 µg/mL DOX overnight at 30 °C. Aliquots of each overnight culture (1 mL) were washed once in H_2_O and diluted to an OD_600_ of 0.8. Cells were 10-fold serially diluted four times in H_2_O in a 96-well plate and 3 µL of cells from each dilution were spotted on YPD agar in the absence and presence of 100 µg/mL DOX. Cells from YPD overnight were spotted on YPD agar and cells from YPD supplemented with 0.05 µg/mL DOX overnight were spotted on YPD agar supplemented with 100 µg/mL DOX. YPD agar plates for box H/ACA ribonucleoprotein complex and SF3b complex GRACE strains were grown for 5 days at 30 °C. YPD agar plates for the *tetO-RHT1*/Δ GRACE strain were grown for 2 days at 30 °C. Spot dilution assays were performed the same way for wild type, deletion mutant, complemented strains, and bypass mutants without DOX.

### Bypass mutant selection

To evolve bypass mutants, single colonies were cultured in 3 mL YPD under shaking conditions at 30 °C for 48 h and 24 h for CaLC10712 and CaLC10720, respectively. Cells were washed once in H_2_O and 10^8^ cells were plated on YPD agar media and incubated at 42 °C for 2 weeks and 5 days, respectively. Colonies were propagated on YPD agar and grown at 30 °C to isolate single colonies. A single colony was used to validate bypass phenotype, isolate genomic DNA extraction, and archive in 25% glycerol for −80 °C storage. For *rht1*Δ/Δ bypass mutants, lineages with restored high temperature sensitivity were obtained from the YPD agar plate during isolation for single colonies (S4 Fig).

### Quantitative reverse transcriptase PCR (qRT-PCR)

To confirm DOX-dependent transcriptional repression, wild-type and GRACE strains were grown in YPD in the absence and presence of 0.05 µg/mL DOX overnight at 30 °C, shaking. The next day, the cultures were sub-cultured to an OD_600_ of 0.2 in 10 mL YPD in the absence and presence of 100 µg/mL DOX and grown for 4 h at 30 °C. GRACE mutants for essential genes were sub-cultured to an OD_600_ between 0.3 and 0.4 in 10 mL YPD in the presence of 100 µg/mL DOX for 4 h at 30 °C. To confirm DOX-dependent transcriptional repression for *YSF3* and *EFG1* GRACE strains used in the filamentation assay, cells were grown as above except on the second day, cells were sub-cultured in YPD in the absence and presence of 20 µg/mL DOX. For ITS1 and ITS2 quantification, overnight cultures of wild-type, *gar1*Δ/Δ, *GAR1A*/*A*, and *gar1*Δ/Δ ByM1 strains were sub-cultured to an OD_600_ of 0.2 in 10 mL YPD and grown for 4 h at 30 °C. Cells were pelleted, washed once with cold H_2_O, flash frozen, and stored at −80°C. Cells were lysed by bead beating four times for 30 s, with one minute on ice between. RNA was extracted using the QIAGEN Rneasy kit and Dnase-treated using the Invitrogen DNA-free DNA removal kit. cDNA synthesis was performed using the iScript cDNA synthesis Kit (Biorad). Standard curve was performed for each pair of primers using wild-type samples to determine the optimal cDNA dilutions for qRT-PCR. cDNAs were diluted 80-fold for expression analysis and 800-fold for ITS1 and ITS2 level quantification. qRT-PCR was performed in technical triplicate with a 10 µL reaction volume in a 384-well plate, using Fast SYBR Green Master Mix (Applied Biosystems) and the BioRad CFX-384 Real Time System. The following cycling conditions were used: 95 °C for 3 min, then 95 °C for 10 s and 60 °C for 30 s, for 40 cycles. The melt curve was completed with the following cycle conditions: 95 °C for 10 s and 65 °C for 10 s with an increase of 0.5 °C per cycle up to 95 °C. The primers used to monitor expression are listed in S8 Data.

### Translation assay

The fluorescent translation assay was performed with the Click-iT HPG Alexa Fluor 488 Protein Synthesis Assay Kit (Thermo Fisher C10428), as previously described [37]. Overnights of SC5314, CaLC10712, and CaLC10713 were sub-cultured to an OD_600_ of 0.2 into 5 mL synthetic defined (SD) medium without amino acids, without ammonium sulfate supplemented with 2% glucose, monosodium glutamate, and uridine and grown at 30 °C, shaking, for 4 h. For positive and negative controls, 1 mL aliquots of SC5314 cells were spun down, and the pellet was resuspended in SD with 100 µM anisomycin or 4 µg/mL fluconazole, and the cultures were incubated under shaking conditions at 30 °C for 10 min. One milliliter aliquots of each sample and drug-treated SC5314 cells were pelleted and resuspended in 1 mL of SD with l-HPG at 1:1,000 dilution. SC5314 cells resuspended in SD without HPG were used as the no-HPG control. All samples were incubated at 30 °C, shaking, for 30 min. Cells were spun down, supernatant removed, and pellets washed once with 1× PBS before being resuspended in 500 µL PBS. Cells were fixed by adding 500 µL 70% ethanol in 1× PBS and rocking for 1 h at room temperature. Cells were then pelleted and washed twice with 3% BSA in PBS. Pellets were resuspended with 500 µL of the reaction cocktail containing the azide fluorophore (prepared according to the manufacturer’s protocol) and incubated for 30 min, shaking in the dark. The samples were washed once with the rinse buffer, pelleted, and resuspended in 20 µL of 1× PBS. Cells were imaged by differential interference contrast microscopy and the EGFP channel on a Zeiss Axio Imager M1 at the same exposure time and analyzed using a CytoFlex flow cytometer (Backman Coulter) to quantify fluorescence intensity. A total of 20,000 events were documented and median fluorescein isothiocyanate value was determined using the CytExpert software.

### Dose-response assays

Drug susceptibility was measured using a modified broth microdilution protocol in 96-well plates (Sarstedt) format, as previously described [92]. Drugs fluconazole, HU, NOC, and MMS were tested in liquid YPD. Cultures were grown in 2-fold dilutions of drug for 48 h as indicated. To assess cidality, 5 µL of mixed cultures were pipetted onto YPD agar following OD_600_ measurements, and the agar plates were incubated at 30 °C for 2 days. Data were displayed quantitatively as heat maps using Java TreeView software (version 1.1.6r4). Reported results are representative of two independent experiments, each performed in technical duplicate.

### Illumina whole-genome sequencing analysis

Genomic DNA was isolated using the phenol-chloroform extraction method as previously described [78]. Libraries were prepared using NEB Ultra II FS DNA Kit and sequenced with Illumina Novaseq X Plus PE150bp to obtain 50–1,000× coverage. Sequencing data were processed as previously described, with minor modifications [77,93]. For genomes with 1,000× coverage, quality control and trimming were performed using fastp (v0.23.4) [94] with minimum qualified phred quality of 20, a 40% limit on unqualified bases, a mean quality requirement of 20, a cut window size of 5, both front and tail cutting, and with correction enabled. For genomes with 50–100× coverage, adaptor sequences and low-quality reads were trimmed using Trimmomatic (v0.39 LEADING:3 TRAILING:3 SLIDINGWINDOW:4:15 MINLEN:36 TORPHRED33) [95]. Trimmed reads were aligned to the *C. albicans* reference genome (SC5314_version_A21-s02-m09-r10) [28] using BWA-MEM (v.0.7.17) [96] with default parameters. Aligned reads were sorted, duplicate reads were marked, and the resulting BAM file was indexed with Samtools (v.1.17) [97] and picard (RRID:SCR_006525, v.2.26.3). The quality of trimmed FASTQ and BAM files was assessed with FastQC (v.0.12.1) [98] and Qualimap (v.2.2.1) [99]. Chromosome copy number changes were visualized using the Yeast Mapping Analysis Pipeline (YMAP v.1.0) [100]. Aligned BAM files were uploaded to YMAP and mapped to the reference genome *C. albicans* SC5314 (A21-S02-m09-r10) with GC-content bias correction for read-depth. Read depth along the *x* axis indicating chromosome position (Chr1–ChrR) was plotted as the log2 ration that was converted to 0–4 as the *y* axis indicating chromosome copy number [100]. Haplotypes are color-coded with gray, magenta, and cyan indicating heterozygous AB, homozygous B, and homozygous A, respectively.

Variant calling and filtering were performed for bypass mutants that failed to revert with Mutect2 and FilterMutectCalls within the GATK package (v.4.6.1.0) [101]. Pairwise variant calling was run for each bypass mutant and its parental strain with the bypass mutant as “tumor” and the parental strain as “normal” using the reference genome *C. albicans* SC5314 (A21-S02-m09-r10). The resulting VCF files were further filtered using bcftools (v.1.21) [97]. Specifically, the parental strain was removed from the VCF file and variant calls were filtered for a quality status of “PASS“ and exclusion of calls in repeat regions as marked in (SC5314 v.A21-s02-m09-r10_features.gff) and 5,000 bp subtelomeric regions. Hard filtering was performed with the following parameters: minimum of 5 supporting reads, at least one supporting read in both forward and reverse direction, minimum alternate allele frequency of 0.2. Identified variants were compared among bypass mutants and only unique calls were kept. Variants were annotated with SnpEff (v.4.3t; database built from SC5314 v.A21-s02-m09-r10, with alternate yeast nuclear codon table) [102], formatted using bcftools [97], and verified in IGV [103,104]. Verified variants were compiled into S3 Table.

### *Galleria mellonella* model of *C. albicans* infection

A *Galleria mellonella* larvae model of infection was performed to test *C. albicans* temperature-dependent virulence as previously described [27]. Briefly, wild type (SC5314), deletion mutants, and complemented strains were grown overnight shaking in YPD at 30 °C. Cells were counted using a hemocytometer to prepare inoculum of 1 × 10^8^ cells per mL. Trypan blue was added to all inoculum stocks to a final concentration of 0.04% as a visual indicator of proper injection in the larvae. Larvae were screened using a weight filter of 230–300 mg and lack of dark spots indicative of injury and visible signs of pupation. Twenty larvae per group were injected in the last left proleg with 1 × 10^6^ cells in a 10 μL volume using a 500 μL gas-tight 1750 series syringe (Hamilton, Ref: CAL81230) with the PB600 repeating syringe dispenser attachment (Hamilton, 83700) for each strain. A group injected with PBS without cells were used as control. Larvae were incubated in petri dishes by treatment group at indicated temperatures and monitored for 144 h for mortality as defined by lack of response to physical touch. Mortality was first checked at 12 h postinfection, then every 4 h between 12 and 24 h postinfection, every 6 h between 24 and 60 h postinfection, and finally every 12 h thereafter until the endpoint of 144 h. Larvae and pupae were euthanized by freezing at −20 °C.

### Cell growth and RNA preparation for *ACT1* splicing assay and RNA sequencing

Overnight cultures of wild-type, *tetO-YSF3*/Δ, and *tetO-PRP19*/Δ strains in YPD in the absence and presence of 0.05 µg/mL DOX were sub-cultured to an OD_600_ of 0.2 and 0.4 in 10 mL YPD in the absence and presence of 20 µg/mL DOX and grown for approximately 3 h at 22 or 37 °C. RNA was extracted, Dnase-treated, and reverse transcribed as described above with the iScript cDNA synthesis Kit (Biorad), which uses a blend of oligo (dT) and random primers to avoid 5′ and 3′ bias. *ACT1* splicing assay was performed as previously described [49]. Briefly, 1 µL of cDNA product was mixed with 0.1 μL of each *ACT1* primer at 100 μM (oLC9781 and oLC10309), 2.5 μL of the supplied 10× reaction buffer, 2 μL of the supplied 2.5 mM dNTP mix, 0.25 μL of TAKARA ExTaq DNA polymerase, and 19 μL of ddH_2_O and subjected to a 30-cycle PCR reaction of 94 °C for 30 s, 52 °C for 45 s, and 72 °C for 60 s. PCR products were run on a 2% agarose gel at 100 V for 60 min, and the SYBR Safe-stained gels were imaged on a ChemiDoc.

Illumina Stranded mRNA Ligation Kit was used to prepare libraries for wild-type and *tetO-YSF3*/Δ RNAs. Libraries were sequenced with Illumina Novaseq X Plus PE150bp to obtain >50 million reads per sample.

### RNA-Seq data processing and mapping

Following quality control by FastQC/0.11.9 [98] using the following command: fastqc -o QC_$OUTNAME $R1 $R2 # “$OUTNAME”_R1t.fastq.gz “$OUTNAME”_R2t.fastq.gz, detected Nextera adapter sequences were trimmed off of the paired-end 150 bp reads using cutadapt/2.10 [105] with the following flags and settings: --cores=0 -b CTGTCTCTTATACACATCT -B CTGTCTCTTATACACATCT -e 0.1 -O 10 -m 16 –output=”$OUTNAME”_R1t.fastq.gz –paired-output= ”$OUTNAME”_R2t.fastq.gz $R1 $R2. Another FastQC quality control was performed after trimming. HISAT2/2.2.1 [106] was used to map the trimmed reads to the *Candida albicans* SC5314 genome version A22 with the following specifications: hisat2 –summary-file “$OUTNAME”_A22_summary.txt –max-intronlen 2000 -x $GENOME2 –rna-strandness FR -1 $R1 -2 $R2 -S “$OUTNAME”_A22.sam –novel-splicesite-outfile “$OUTNAME”_novelSS.txt. Unmapped reads were removed, and the generated output of the mapped reads was converted into tdf and bedgraph files using SAMtools/1.16/1 [107], BEDtools/2.30.0 [108] and IGVtools [104] for IGV visualization and further analyses.

### Expression analysis and principal component analysis

Read counts per gene for differential gene expression analysis were obtained using a custom bash script involving SAMtools/1.9, BEDTools/2.27.1 and a custom R script, as described in Lash and colleagues [49]. Specifically, the resulting bam files from mapping were split into first and second reads with SAMtools view -h -b -f 0x40 $BAM > r1.bam/ 0x80 $BAM > r2.bam. Following intersection with the genome annotation by bedtools intersect, reads overlapping with annotated features were counted using awk and bash commands. Resulting files contained reads per gene, as well as total mapped reads (including intergenic regions). Differential gene expression analysis was performed with custom R scripts using the DESeq2 package [109] from Bioconductor. DESeqDataSetFromMatrix was used to create the DESeqDataSet object to run DeSeq, comparing every condition with each other (S2 Data). Principal component analysis was performed by using vst and plotPCA functions. To create custom colors and shapes based on condition, ggplot2 [110] was used for better visualization of the PCA plot.

For the analysis towards Figs 3H and S6E, intron-containing gene expression values were adjusted to consider exclusively the CDS for gene expression and excluding reads covering introns that are expected to differ strongly in counts due to decreased pre-mRNA splicing upon *YSF3* depletion. First, the reads per intron were calculated using the same approach as for obtaining reads per gene, except for using the intron annotation for bedtools intersect. The obtained intron counts, as well as the total gene counts were normalized for library size by dividing by the number of total mapped reads for each sample. Following normalization, the average counts were calculated between replicates of the same condition. Next, the normalized, averaged number of reads per intron was subtracted from the number of reads per (intron-containing) gene to obtain ‘reads per exon’, to get a measure for expression of transcripts that could actually be subject to translation. Combined exon length was calculated by subtracting the intron length from gene length. Then, reads per kilobase (RPK) values were calculated by dividing number of reads per exon by the exon length in kilobases. To obtain spliced and unspliced intron-containing gene expression, RPK values were multiplied by the respective IR fraction for that gene and condition (S5 Data). Genes with multiple introns were excluded from this analysis.

### IR quantification

For splicing and IR quantification, the intron annotation used contained annotated introns from CGD, including 5′UTR introns. Analysis was carried out as in Lash and colleagues [49] using custom bash and R scripts. Specifically, bam files were split into forward and reverse reads again using samtools view -h -b -f 0x40 $BAM > r1.bam/0x80 $BAM > r2.bam. Afterwards, reads were overlapped with the annotated introns maintaining strand information using bedtools intersect. A minimum overlap of three bases was required to consider a read overlapping. The block count shows the number of continuous aligned segments in a read. Reads with a block count of 1, indicating alignment without gaps, were considered unspliced and were further categorized into intronic or unspliced, depending on the start and end position of the read; If the block count is > 1, indicating a gap in the aligned read and thus an exon-exon junction, it was classified as spliced. Split reads were further divided into spliced (exactly matching annotated intron) or alternatively spliced (no perfect match). For each intron, reads for each of the categories were counted. For each intron, IR fraction was calculated by dividing the number of unspliced reads by the sum of the number of unspliced and 2 × the number of spliced reads (S3 and S4 Data). We introduced factor 2 for “spliced” junction reads to account for the aspect that one unspliced transcript can give rise to two “unspliced” reads, but a spliced transcript can only yield one “spliced” read. A minimal read cutoff of 20 was required for further analysis. IR difference was calculated as the difference between the fraction of IR of each induced (+ DOX) and uninduced (− DOX) sample.

### Branchpoint sequence identification

To computationally identify likely BPSs in *C. albicans* introns we made use of previously annotated 7-nt branchpoint motifs in *S. cerevisiae* and *C. albicans* [52,111]. First, we calculated the frequency, position weight, and logOdds matrix from the frequency of nucleotides at each position within the 7-nt motif and the average nucleotide frequency across *S. cerevisiae* introns using a custom R script and the Biostrings package. Using the *S. cerevisiae* LogOdds matrix we calculated a branchpoint score for each 7-nt running window across *C. albicans* introns by summing up LogOdds matrix entries at the respective motif position (column) and base identity (row). To select likely BPSs we required a score above 4. Three introns did not have a score above 4, filtering for a score greater than 0 yielded a likely branchpoint for all three introns. Overall, 333 introns showed the consensus BPS of 5′ UACUAAC 3′ and 76 introns showed slight variations of that motif (non-consensus BPSs) (S3 Data). Seventy-nine introns had more than one possible branchpoint, whereas we considered the BPSs matching the median distance between BPS and 3′ SS best as the primary branchpoint for our analysis. The median distance between BPS and 3′ SS is 24 nts and matches previous estimates [51]; the distance includes the 7-nt BPS. Introns were categorized by their BP motif (consensus/non-consensus) and into four ranges of BPS to 3′ SS distance using quantile to ensure equal distribution of the data.

### Intron feature analysis

Intron lengths were obtained through the start and end intron coordinates. As intron lengths underlie a bimodal distribution (S6C Fig), we determined a cutoff between short and long introns by analyzing the distribution of intron lengths in our dataset using kernel density estimation (density() function in R). The density plot revealed two prominent peaks, corresponding to two major intron length modes. We used the findpeaks() function (pracma package) to identify these peaks and then determined the local minimum (valley) in the density curve between them. This valley was used as a threshold to classify introns into “short” (≤valley position) and “long” (>valley position). The first 6 nucleotides of an intron were considered for the 5′SS analysis and the last 3 intronic nucleotides to call 3′SS sequences. The conserved 5′SS motif consists of the sequence GUAUGU (GTATGT in the DNA sequ“nce),”and we defined the 3′SS consensus as the most conserved TAG motif, while also including CAG and AAG, which are common 3′SSs in *C. albicans* as well [51]. We found that all introns for which we measured IR by RNA-seq had a consensus 3′SS, with TAG being the most abundant comprising 298 introns (73%), AAG was found in 58 introns (14%) and 50 introns had a CAG 3′SS (12%). There was some variation within the 5′ SS, while most of them (336 introns) had the full consensus sequence, which is almost 83%, and 62 introns had only the strongly conserved first three nucleotides GTA of the motif, which in combination makes up almost all introns (398/98%). Thus, we categorized the SSs into consensus or variable 5′ SS (S4 Data). When plotting the Δ IR fraction (IR difference) for the categories intron length, BP motif, SS motif and BP–3′SS distance, we verified that the observed effects are consistent when all other variables are kept constant (optimal motifs/distance). Similar analysis for GC-content, polypyrimidine tract length and CU-fraction within the polypyrimidine tract did not produce significant differences in IR upon Ysf3 depletion.

### GO analysis of differentially expressed genes

As in Lash and colleagues [49] GO term enrichment was performed using the combined GO annotations from CGD (https://geneontology.org/, version from 08/10/2023, gaf-version: 2; and version from 17/01/2024, gaf-version: 2.2) and GO annotations from *S. cerevisiae* orthologs (https://geneontology.org/, version from 18/01/2024, gaf-version: 2.2). *S. cerevisiae* orthologous genes were matched according to the information on CGD. GO term annotations were extended to all parents of individual GO terms using the “get_parent_nodes” function from the GOfuncR package. Occurrences of GO terms were counted in each gene group of interest (up- or down-regulated genes upon *YSF3* depletion at 22 or 37 °C, respectively) and compared to their frequency in the gene background. For GO term enrichment among groups of differentially expressed genes we distinguished between upregulated genes (log2-fold change of differential expression > 0.5 and adjusted *P*-value < 0.00001, 592 genes at 22 °C and 239 genes at 37 °C) and downregulated genes (log2-fold change of differential expression >0.5 and adjusted *P*-value <0.00001, 616 genes at 22 °C and 342 genes at 37 °C). In this case, the gene background was formed by all genes that were quantified as expressed in the RNA-seq data. To assess the significance of GO term enrichment, we performed the Fisher’s Exact Test and multiple testing correction (Bonferroni correction). A *P*-value cutoff of <0.01 was used to call positively enriched GO terms for “Biological Process.” Enriched GO terms for “Biological Process,” “Molecular Function,” and “Cellular Component” across up- and down-regulated genes are listed in S6 Data.

### Morphogenesis assay

To determine the impact of *YSF3* on morphogenesis, CaSS1, *YSF3,* and *EFG1* GRACE strains were grown in YPD in the presence and absence of 0.05 µg/mL DOX overnight at 30 °C, shaking, and sub-cultured to an OD_600_ of 0.2 in YPD supplemented with 10% newborn calf serum in the presence and absence of 20 µg/mL DOX at 37 °C, shaking, for 6 h. Cells were then imaged on a Zeiss Axio Imager MI.

To determine the impact of *RHT1* on morphogenesis, overnight cultures of SC5314, CaLC10720, and CaLC10721 were grown in YPD at 30 °C, shaking, and sub-cultured to an OD_600_ of 0.2 in YPD. SC5314 cells treated with 25 mM HU and 50 µM NOC were used as controls. Cultures were grown at 30 °C for 4 h before imaging on a Zeiss Axio Imager MI.

### Immunoprecipitation

Immunoprecipitation of GFP-tagged Rht1 was performed to assess *RHT1-GFP* expression, as previously described [112]. Briefly, overnight cultures were sub-cultured to an OD_600_ of 0.2 in 50 mL YPD and grown for 4 h at 30 °C. Cells were pelleted and washed in 1 mL of F300 buffer (50 mM HEPES-KOH pH 7.2, 300 mM KOAc, and 10% glycerol) and stored at −80 °C. To prepare whole cell lysate, each pellet was resuspended in 1 mL F300 and disrupted by bead-beating (2 min on; 1 min rest on ice) for three cycles. Crude lysates were mixed with a half-volume of F300 supplemented with 0.06% IGPAL-CA630 and sonicated (20 s  × 4) at 30% amplitude using a 3-mm diameter probe. Sonicated lysates were treated with ~30 U Benzonase (Millipore) for 20 min after the addition of MgCl_2_ to the final centration of 2 mM and clarified by centrifugation at 20,000 *g* for 20 min at 4 °C. Total protein (~6 mg) in supernatant was incubated with 20 µL of anti-GFP magnetic beads (GTMA-20; Chromotek) for 90 min on a revolving rotator. Bead-bound proteins were captured using a magnetic rack and washed with F300 + 0.02% IGEPAL-CA630 × 3 before boiling in 30 µL 1 × SDS-PAGE loading buffer. All eluted proteins were resolved on 10% precast SDS-gels and blots probed with HRP-conjugated anti-GFP antibody (118144600012; Roche; 1:3,000) followed by HRP-conjugated goat anti-mouse secondary antibody (170-65169; Bio-Rad; 1:5,000). All F300 buffers were supplemented immediately prior to use with 1× PhosSTOP (Roche) and 1× Complete inhibitor cocktails (Roche) to inhibit phosphatase and proteinase activity.

### Mass spectrometry

AP-MS experiments were performed as previously described [27]. Briefly, Rht1-GFP (CaLC10748) and Eno1-GFP (CaLC4449) strains were grown overnight at 30 °C in YPD. Stationary phase cultures were diluted to an OD_600_ of 0.1 in 1 L YPD and grown to an OD_600_ between 0.6 and 0.8. Cells were harvested at 3,000 rpm for 30 min at 4 °C, washed with ice-cold water, and snap-frozen in liquid nitrogen bath. For protein extraction, samples were diluted 1:1 by weight in lysis buffer (50 mM HEPES [pH 7.5], 150 mM NaCl, 5mM EDTA, 5mM DTT, 0.1% NP-40, 1× ROCHE protease inhibitor cocktail tablet [Roche Diagnostics, Mississauga, ON, Canada]) and vortexed with glass beads (0.5 mm) for 4 × 1 min. Lysates were collected by stacked transfer for 5 min at 100*g* with a 18-gauge needle and clarified by centrifugation at 16,110*g* for 20 min in 4 °C in a microcentrifuge.

To affinity purify the GFP-interacting proteins, we used 25 µL of GFP-TRAP_M magnetic beads (ChromoTek, Martinsried, Germany) for each 1 L culture. Beads were equilibrated three times with 1 mL of lysis buffer (50 mM HEPES [pH 7.5], 150 mM NaCl, 5 mM EDTA, 5 mM DTT, 0.1% NP-40, 1× ROCHE protease inhibitors [Roche Diagnostics, Mississauga, ON, Canada]). The protein extract was then added to the resin and rotated for 2 h at 4 °C. The beads were magnetized then washed with 1 mL of lysis buffer and then 1 mL of wash buffer (20 mM Tris [pH 8.0], 2 mM CaCl_2_). The dried beads were resuspended in 20 mM Tris-HCl, and incubated with 0.5 μg trypsin (Sigma, T7575) with rotation at 37 °C overnight (~15 h). The samples were digested on-bead with 0.75 μg trypsin (0.2 μg/μL in 20 mM Tris-HCl, pH 8.0) at 37 °C overnight with rotation. Magnetization and digestion were repeated once more with a shorter incubation of 3–4 h without rotation. Formic acid was added to a final concentration of 2% and stored at −40 °C until used for MS.

AP-MS samples and controls were analyzed by MS in two biological replicates. 1/16th of each sample was acquired in data-dependent acquisition (DDA) via LC–MS/MS. Samples were loaded onto Evotip Pure cartridges and eluted from the Performance EV1109 column (8 cm × 150 µm with 1.5 µm beads), heated at 40 °C, with the 60SPD gradient on an Evosep One. The column was coupled to the timsTOF Pro 2 using a 20 µm diameter emitter tip (Bruker 1811107). Peptides were eluted from the column with an acetonitrile gradient generated by an Eksigent ekspert nanoLC 425 and analyzed on a TripleTOF 6600 instrument (AB SCIEX, Concord, Ontario, Canada). The total DDA protocol is 22 min. The MS1 scan had a mass range of 100–1,700 Da in PASEF mode. TIMS settings were accumulation and ramp time of 100 ms (with 4 PASEF ramps and active exclusion at 0.4 min), and within the mobility range (1/K0) of 0.85–1.3 V·s/cm^2^. This was at a cycle time of 0.53 s. The target intensity was set to 17,500 and the intensity threshold set to 1,750. 1+ ions are excluded from fragmentation using a polygonal filter. The auto calibration was off.

MS data generated were stored, searched, and analyzed using the ProHits laboratory information management system (LIMS) platform and searched using MSFragger v4.1 [113] against the *C. albicans* UniProt database (UP000000559) acquired from NCBI, supplemented with common contaminants, decoy entries were added. Acetylated protein N-term and oxidated methionine were set as variable modifications. Precursor mass tolerance was set to 20 ppm on either side. Fragment mass tolerance was set to 20 ppm. Enzymatic cleavage was set to trypsin with 2 missed cleavages. MSBooster and Percolator were turned on. Percolator required a minimum probability of 0.5 and did not remove redundant peptides. The target-decoy competition method was used to assign q-values and PEPs. For ProteinProphet, the maximum peptide mass difference was set to 30 ppm. When generating the final report, the protein FDR filter was set to 0.01. FDR was estimated by using both filtered PSM and protein lists. Razor peptides were used for protein FDR scoring. All other parameters were default. Results were analyzed with SAINTexpress (v3.3) [114] with Eno1-GFP samples as negative controls. A total spectral count greater than or equal to 20 and a BFDR value equal to 0 were required for proteins to be classified as significant interaction partners (S7 Data). Data has been deposited as a complete submission to the MassIVE repository (https://massive.ucsd.edu/ProteoSAFe/static/massive.jsp) and assigned the accession number MSV000097442. The ProteomeXchange accession is PXD062215. The dataset is currently available for reviewers at ftp://MSV000097442@massive.ucsd.edu. Please login with username MSV000097442_reviewer; password: k5Zd8dQmZ8qZz70E. The dataset will be made public upon acceptance of the manuscript. Cytoscape [115] was used to visualize high-confidence protein interactors identified by AP-MS.

### E-test strip and disc diffusion assays

E-test strip assay and disc diffusion assays were performed to test fluconazole tolerance for *C. albicans* strains, as previously described [116]. Wild type, *rht1*Δ/Δ mutant, complemented strain, ByM1, ByM1R, and ByM10s were grown in liquid YPD at 30 °C overnight, washed once with sterile H_2_O, and diluted to a cell density of 10^8^ cells/mL. About 100 µL cells were spread on YPD agar using a sterile cotton swab. Fluconazole E-test strip (bioMérieux) or a 0.5 cm precut filter disc was placed at the center of the YPD agar plates. A volume of 5 µL of 10 mg/mL fluconazole stock was spotted on the filter disc. Plates were incubated upside down at 30 or 35 °C and imaged on a ChemiDoc after 3 days.

## Supporting information

S1 Fig Screening of expanded GRACE mutant collection to identify genes for thermal adaptation in *Candida albicans.* **A)** Summary of GRACE mutant collection expansion based on gene ontology annotation. Pie chart with four concentric rings visualizes the relative proportion of genes associated with 22 GO Slim “biological processes” terms from *Candida* Genome Database (CGD). Rings represent gene ontology summaries of the 2,326 genes in GRACEv1, 891 genes in GRACEv2, 1,235 genes in GRACEv3, and 1,775 remaining genes in the *C. albicans* genome for which GRACE mutants have yet to be constructed. **B)** Schematic of screening of the GRACE library at 30 and 39 °C. **C)** Venn diagram showing the overlap of genes that were scored on average >1 in the presence at DOX at 30 and 39 °C. The data underlying S1C Fig can be found in S1 Data. **D)** Schematic of screening of the prioritized 947 GRACE strains at different temperatures. Created in BioRender. Fu, C. (2025) https://BioRender.com/6hhyxa4.
(TIFF)

S2 Fig Quantification of transcript levels of prioritized GRACE strains. Reverse transcriptase quantitative PCR (RT-qPCR) results to confirm **A)** relative expression of indicated transcripts for all GRACE strains and **B)** ITS1 levels in wild type and GRACE strains for *GAR1* and *CBF5*. Wild-type strain and the GRACE strains were grown overnight in YPD with and without 0.05 µg/mL DOX and sub-cultured to an OD_600_ of 0.2 in YPD with and without 100 µg/mL DOX. Cultures were grown at 30 °C for 4 h. RNA was extracted and cDNA was synthesized. Relative level of expression for each gene was normalized to *ACT1*. Bar graphs depict the mean ± SD of technical triplicate (** *p* ≤ 0.01, *** *p* ≤ 0.001, **** *p* ≤ 0.0001, One-way ANOVA Bonferroni’s correction). Each experiment was performed in biological duplicate with consistent results. The data underlying this Figure can be found in S1 Data.
(TIFF)

S3 Fig *Candida albicans* Box H/ACA ribonucleoprotein complex members show differential importance for fitness at different temperatures. Wild-type and GRACE strains were grown overnight in YPD with or without 0.05 µg/mL DOX and spotted in 10-fold dilutions starting from an OD_600_ of 0.8 onto YPD with or without 100 µg/mL DOX. GRACE strain for *HSP90* was included as an essential gene control. Plates were incubated at indicated temperatures and imaged after 5 days.
(TIFF)

S4 Fig Schematic of bypass mutant selection strategy for A) *gar1*Δ/Δ and B) *rht1*Δ/Δ mutant strains. Created in BioRender. Fu, C. (2025) https://BioRender.com/yma9nph.
(TIFF)

S5 Fig *Candida albicans* SF3b complex members show differential importance for fitness and *YSF3* is important for filamentation. **A)** Spotting assay was performed as described in S3 Fig. **B)** Strains were grown overnight in YPD with or without 0.05 µg/mL DOX at 30 °C and sub-cultured to an OD_600_ of 0.2 in YPD supplemented with 10% newborn calf serum with and without 20 µg/mL DOX and grown at 37 °C for 6 h. Scale bar represents 50 µm. **C)** Reverse transcriptase quantitative PCR (RT-qPCR) was performed as described in S2 Fig except sub-culture in YPD with or without 20 µg/mL DOX. Relative levels of expression for *YSF3* and *EFG1* were normalized to *ACT1*. Bar graphs depict the mean ± SD of technical triplicate (** *p* ≤ 0.01, *** *p* ≤ 0.001, **** *p* ≤ 0.0001, One-way ANOVA Bonferroni’s correction). Each experiment was performed in biological duplicate with consistent results. The data underlying S5C Fig can be found in S1 Data.
(TIFF)

S6 Fig Ysf3 modulates splicing efficiency. **A)** RNA-seq read coverage over the *ACT1* gene of WT and *tetO−YSF3*/Δ strains with and without DOX at 22 °C (top) and 37 °C (bottom), complementary to the *ACT1* splicing assay (Fig 3C). **B)** The cumulative distribution shows the IR fraction of 389 introns passing the read count cutoff in all samples. Compared to Fig 3E the x-axis is reduced to 0–0.3 to better visualize differences around the sample cluster that is not *YSF3*-depleted. The data underlying S6B Fig can be found in S3 Data. **C)** Bimodal intron length distribution. For analysis of IR dependent on length, we group introns into “short” and “long” based on the minimum at 287 nt (dashed line). The data underlying S6C Fig can be found in S3 Data. **D)** IR difference of wild type (WT) and *tetO*-*YSF3*/Δ strain without and with DOX at 37 °C of introns grouped by their intron length, BP motif, splice site, and BP-3′SS distance, respectively (see Fig 3G for the 22 °C data). Top numbers indicate introns per group. BP-3′SS distance groups have equal bin size. Significances were obtained using Wilcoxon rank-sum test (intron length, BP motif, splice site) and paired Wilcoxon rank-sum test with Benjamini–Hochberg correction (BP–3′SS distance). The data underlying S6D Fig can be found in S4 Data. **E)** Pie charts of unspliced or spliced intron-containing gene expression in tetO-*YSF3*/Δ without and with DOX at 37 °C. The data underlying S6E Fig can be found in S5 Data. **F)** Cumulative distribution of log2-fold change in gene expression between tetO-*YSF3*/Δ with and without DOX for the indicated gene groups at 37 °C. Significances are relative to the ‘all’ genes (Kolmogorov–Smirnoff test). The data underlying S6F Fig can be found in S5 Data. **G)** Significantly enriched ‘Biological process’ GO terms among downregulated genes in *tetO−YSF3*/Δ with DOX at 22 °C. The data underlying S6G Fig can be found in S6 Data. Numerical data underlying this Figure can be found in S1 Data.
(TIFF)

S7 Fig Fitness scores for Rht1-interacting proteins. **A)** To confirm GFP-tagged Rht1 functionality, strains were grown overnight in YPD and spotted in 10-fold dilution starting from an OD_600_ of 0.8 onto YPD. Plates were incubated at indicated temperatures and imaged after 2 days. **B)** Detection of GFP-tagged Rht1 by IP-western blot. Wild-type and a GFP-tagged Iml3 strains were used as negative and positive controls. Strains were sub-cultured to an OD_600_ of 0.2 in YPD and grown at 30 °C for 4 h. Total proteins were extracted. GFP-tagged proteins were purified using anti-GFP magnetic beads, separated on SDS-gel, and blotted for detection of GFP signal with anti-GFP antibody. The Rht1-GFP strain was repeated twice. Full-length Rht1-GFP and Iml3-GFP are predicted to be 57.5and 58.3 kDa in size, respectively. **C)** Secondary screen results of GRACE strains for Rht1-interacting proteins were analyzed. Gene groups are color-coded as in Fig 5C. DOX scores across the six temperatures are color-scaled as described in Fig 1D. Genes in clusters 2 and 6 are highlighted in orange, with those located on Chromosomes 1 and 2 shown in a darker shade. Source blot images underlying S7B can be found in S1 File Raw_Images.
(TIFF)

S8 Fig Rht1 affects fluconazole activity at 35 °C. **A)** Day 5 images for Fig 5F. **B)** Day 5 images for Fig 5G.
(TIFF)

S1 Table Strains used in this study. (DOCX)

S2 Table Plasmids used in this study. (DOCX)

S3 Table Whole-genome sequencing variant calls. Verified variants for *gar1*Δ/ΔByM1 and *rht1*Δ/ΔByM10 were provided in the table.
(DOCX)

S1 Data Source data for Figs 1B–1E, 2C, 2D, 2G, 3D–3J, 5B, 5D, 5E, S2A, S2B, S5C, and S6B–S6G.
(XLSX)

S2 Data Gene expression based on DeSeq2 analysis. (XLSX)

S3 Data Mean intron retention fraction and branchpoint information of analyzed introns across all samples. (XLSX)

S4 Data Δ IR fraction ± DOX and intron feature information of analyzed introns of WT and *tetO-YSF3*/Δ strains. (XLSX)

S5 Data Intron-containing gene expression based on splicing state. (XLSX)

S6 Data Enriched GO terms in the group of up- and down-regulated genes upon depletion of *YSF3* at 22 and 37 °C. (XLSX)

S7 Data Mass spectrometry analysis; C6_00110c interactome. (XLSX)

S8 Data Primers used in this study. (XLSX)

S1 File Raw Images: Raw gel and blot images for Figs 3C and S7B. (PDF)

## Abbreviations

AP-MS: affinity purification coupled with mass spectrometry
BFDR: Bayesian False Discovery Rate
BPS: branchpoint sequence
CDS: coding sequence
CGD: *Candida* Genome Database
DDA: data-dependent acquisition
DOX: doxycycline
FDR: false discovery rate
GO: gene ontology
GRACE: Gene Replacement and Conditional Expression
HET: heterozygous
HPG: L-homopropargylglycine
HU: hydroxyurea
IR: intron retention
MMS: methyl methanesulfonate
NAT: nourseothricin
NMD: nonsense-mediated decay
NOC: nocodazole
qRT-PCR: quantitative reverse transcriptase PCR
RPK: reads per kilobase
rRNA: ribosomal RNA
SD: synthetic defined
SS: splice site
TM: template modeling
YPD: yeast extract peptone
