# Supplementary figures and images for: Expansion of the functional genomics GRACE library reveals genes relevant for temperature-dependent fitness in Candida albicans
Source: PLoS Biol. 2025 Oct 17;23(10):e3003409. doi: 10.1371/journal.pbio.3003409 (PMC12533916; doi:10.1371/journal.pbio.3003409)

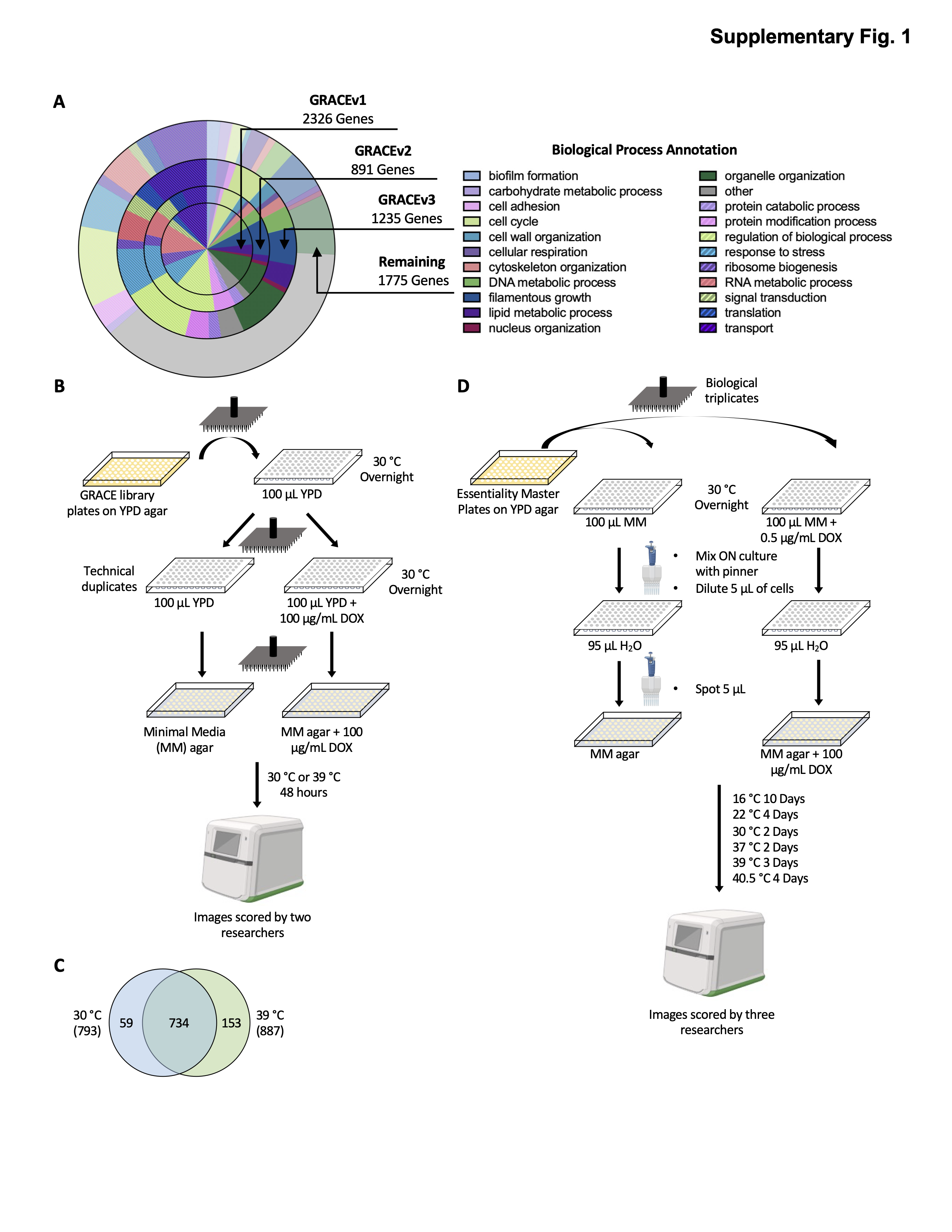

Supplement: S1 Fig — A) Summary of GRACE mutant collection expansion based on gene ontology annotation. Pie chart with four concentric rings visualizes the relative proportion of genes associated with 22 GO Slim “biological processes” terms from Candida Genome Database (CGD). Rings represent gene ontology summaries of the 2,326 genes in GRACEv1, 891 genes in GRACEv2, 1,235 genes in GRACEv3, and 1,775 remaining genes in the C. albicans genome for which GRACE mutants have yet to be constructed. B) Schematic of screening of the GRACE library at 30 and 39 °C. C) Venn diagram showing the overlap of genes that were scored on average >1 in the presence at DOX at 30 and 39 °C. The data underlying S1C Fig can be found in S1 Data. D) Schematic of screening of the prioritized 947 GRACE strains at different temperatures. Created in BioRender. Fu, C. (2025) https://BioRender.com/6hhyxa4. (TIFF) [file pbio.3003409.s001.tiff]

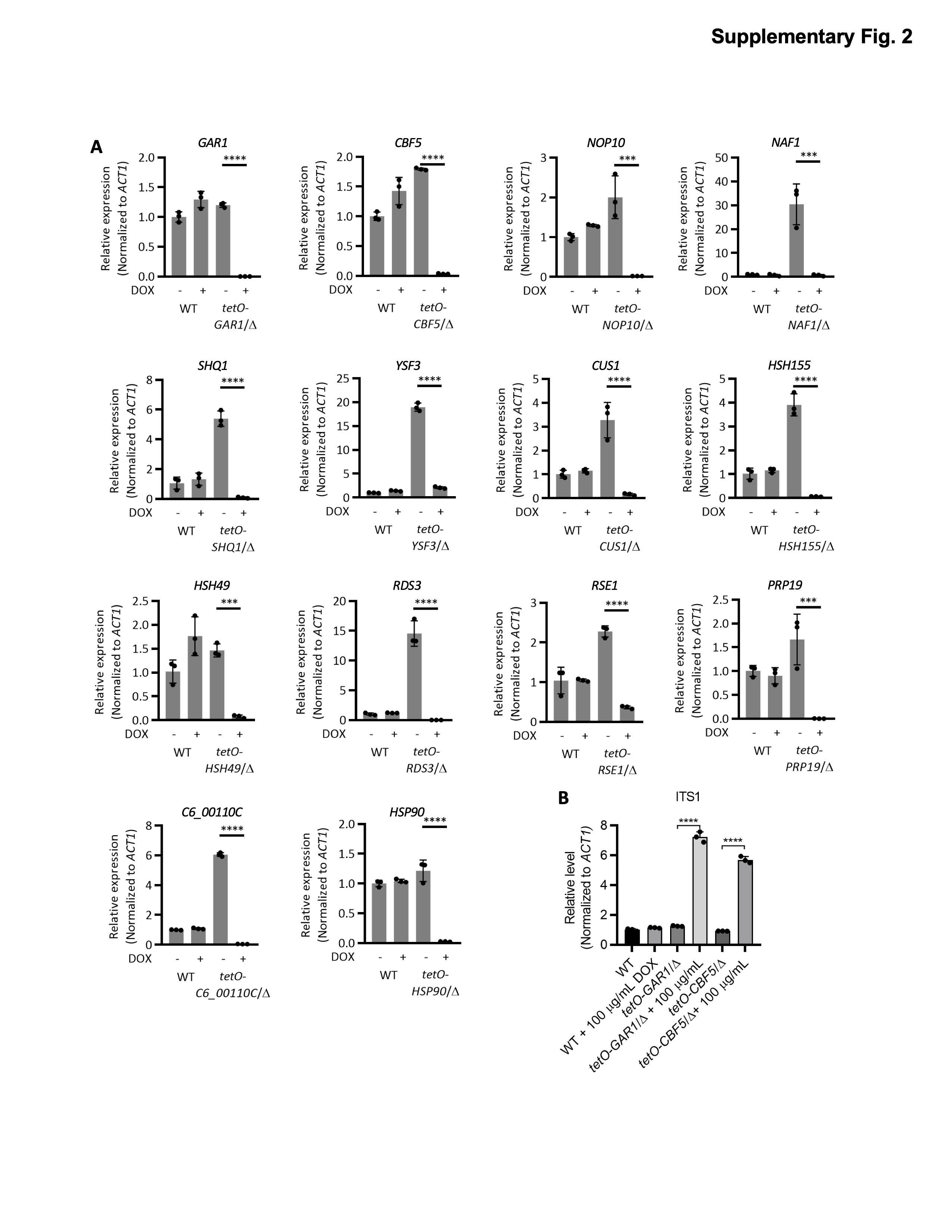

Supplement: S2 Fig — Reverse transcriptase quantitative PCR (RT-qPCR) results to confirm A) relative expression of indicated transcripts for all GRACE strains and B) ITS1 levels in wild type and GRACE strains for GAR1 and CBF5. Wild-type strain and the GRACE strains were grown overnight in YPD with and without 0.05 µg/mL DOX and sub-cultured to an OD600 of 0.2 in YPD with and without 100 µg/mL DOX. Cultures were grown at 30 °C for 4 h. RNA was extracted and cDNA was synthesized. Relative level of expression for each gene was normalized to ACT1. Bar graphs depict the mean ± SD of technical triplicate (** p ≤ 0.01, *** p ≤ 0.001, **** p ≤ 0.0001, One-way ANOVA Bonferroni’s correction). Each experiment was performed in biological duplicate with consistent results. The data underlying this Figure can be found in S1 Data. (TIFF) [file pbio.3003409.s002.tiff]

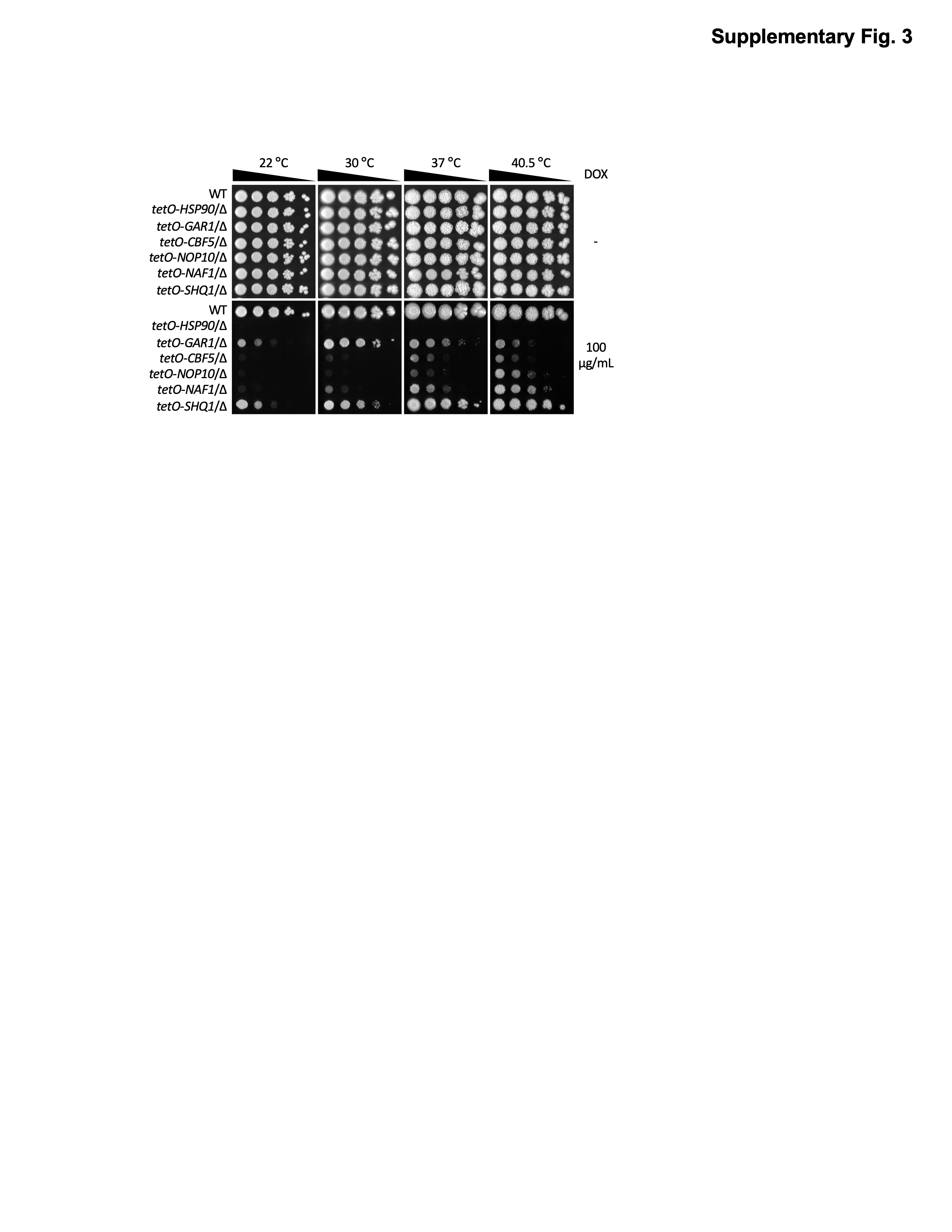

Supplement: S3 Fig — Wild-type and GRACE strains were grown overnight in YPD with or without 0.05 µg/mL DOX and spotted in 10-fold dilutions starting from an OD600 of 0.8 onto YPD with or without 100 µg/mL DOX. GRACE strain for HSP90 was included as an essential gene control. Plates were incubated at indicated temperatures and imaged after 5 days. (TIFF) [file pbio.3003409.s003.tiff]

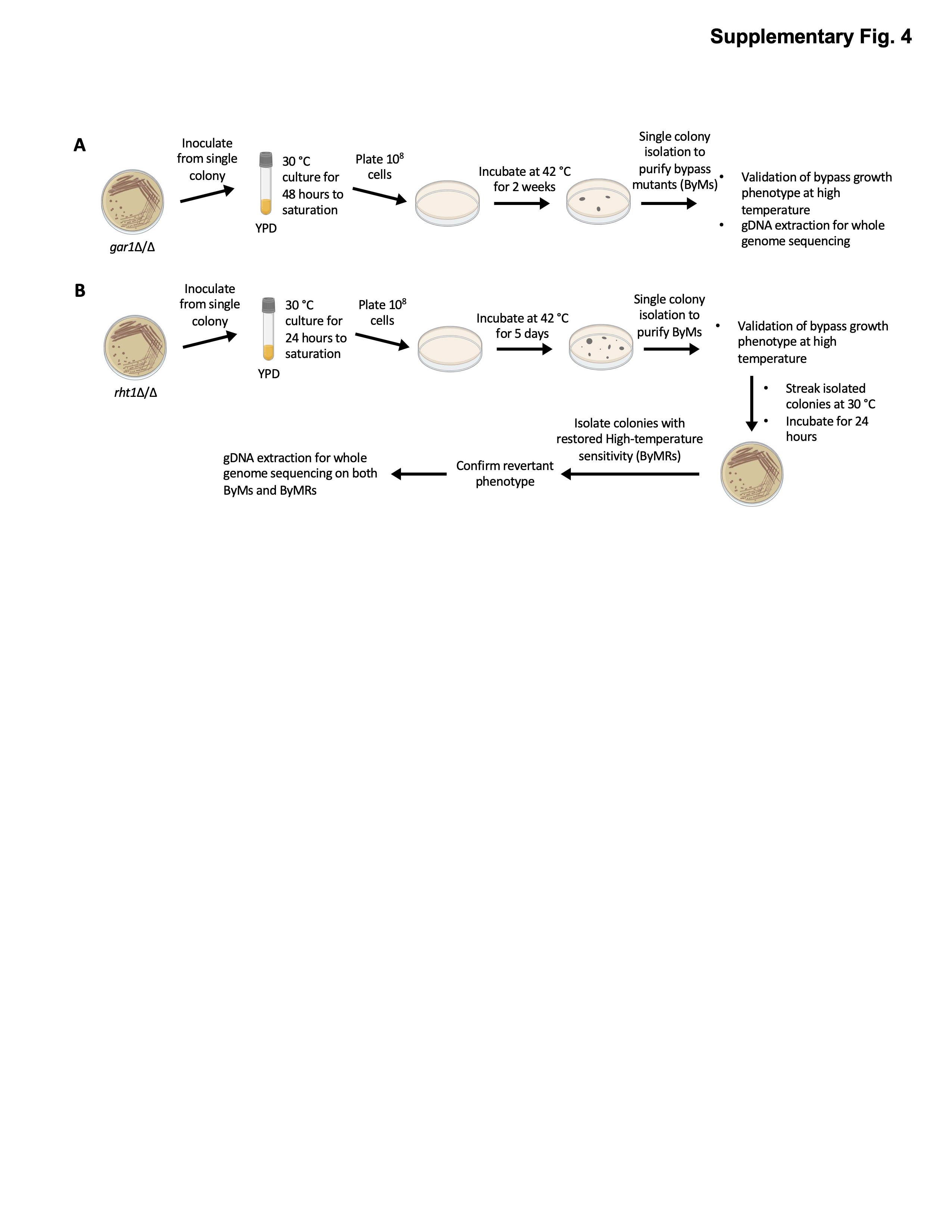

Supplement: S4 Fig — Created in BioRender. Fu, C. (2025) https://BioRender.com/yma9nph. (TIFF) [file pbio.3003409.s004.tiff]

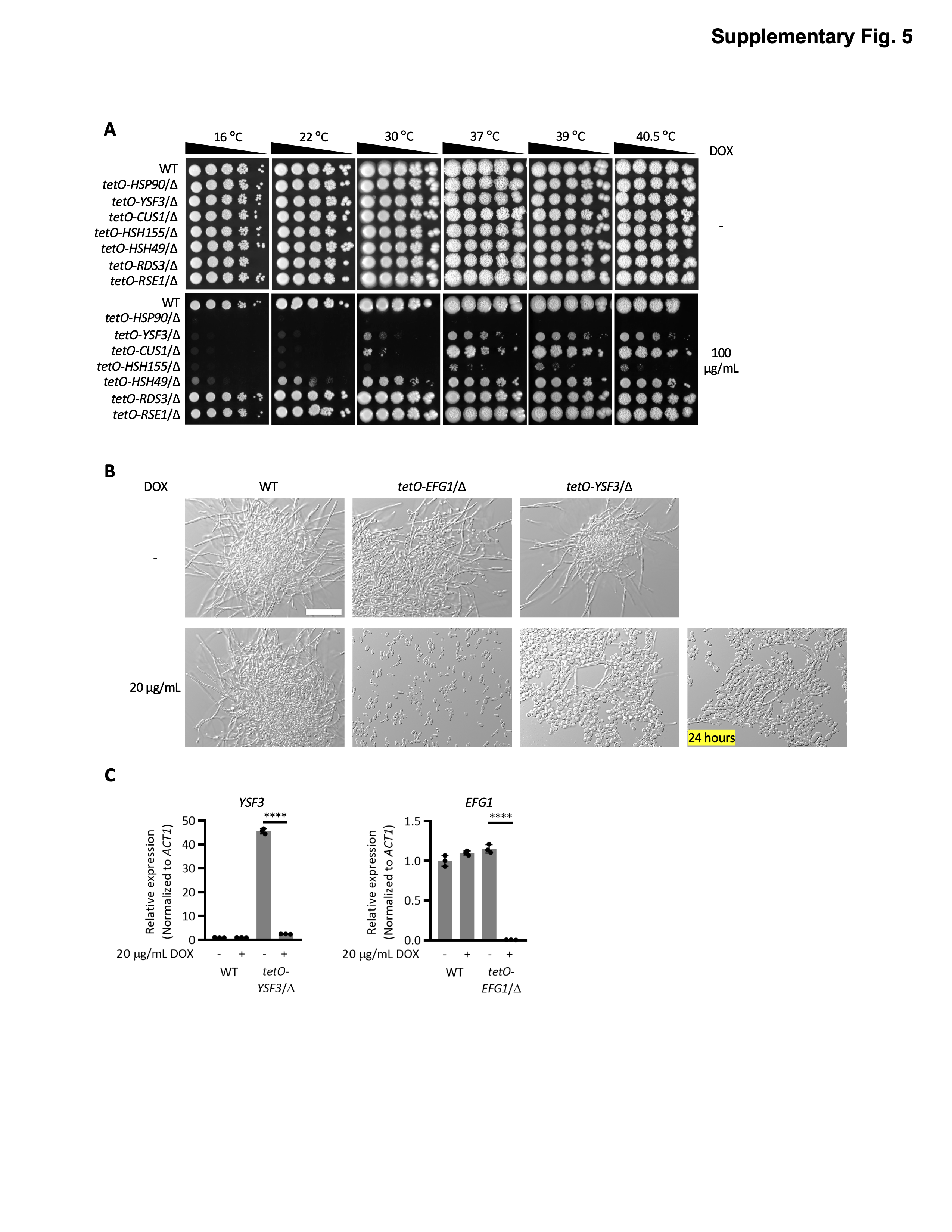

Supplement: S5 Fig — A) Spotting assay was performed as described in S3 Fig. B) Strains were grown overnight in YPD with or without 0.05 µg/mL DOX at 30 °C and sub-cultured to an OD600 of 0.2 in YPD supplemented with 10% newborn calf serum with and without 20 µg/mL DOX and grown at 37 °C for 6 h. Scale bar represents 50 µm. C) Reverse transcriptase quantitative PCR (RT-qPCR) was performed as described in S2 Fig except sub-culture in YPD with or without 20 µg/mL DOX. Relative levels of expression for YSF3 and EFG1 were normalized to ACT1. Bar graphs depict the mean ± SD of technical triplicate (** p ≤ 0.01, *** p ≤ 0.001, **** p ≤ 0.0001, One-way ANOVA Bonferroni’s correction). Each experiment was performed in biological duplicate with consistent results. The data underlying S5C Fig can be found in S1 Data. (TIFF) [file pbio.3003409.s005.tiff]

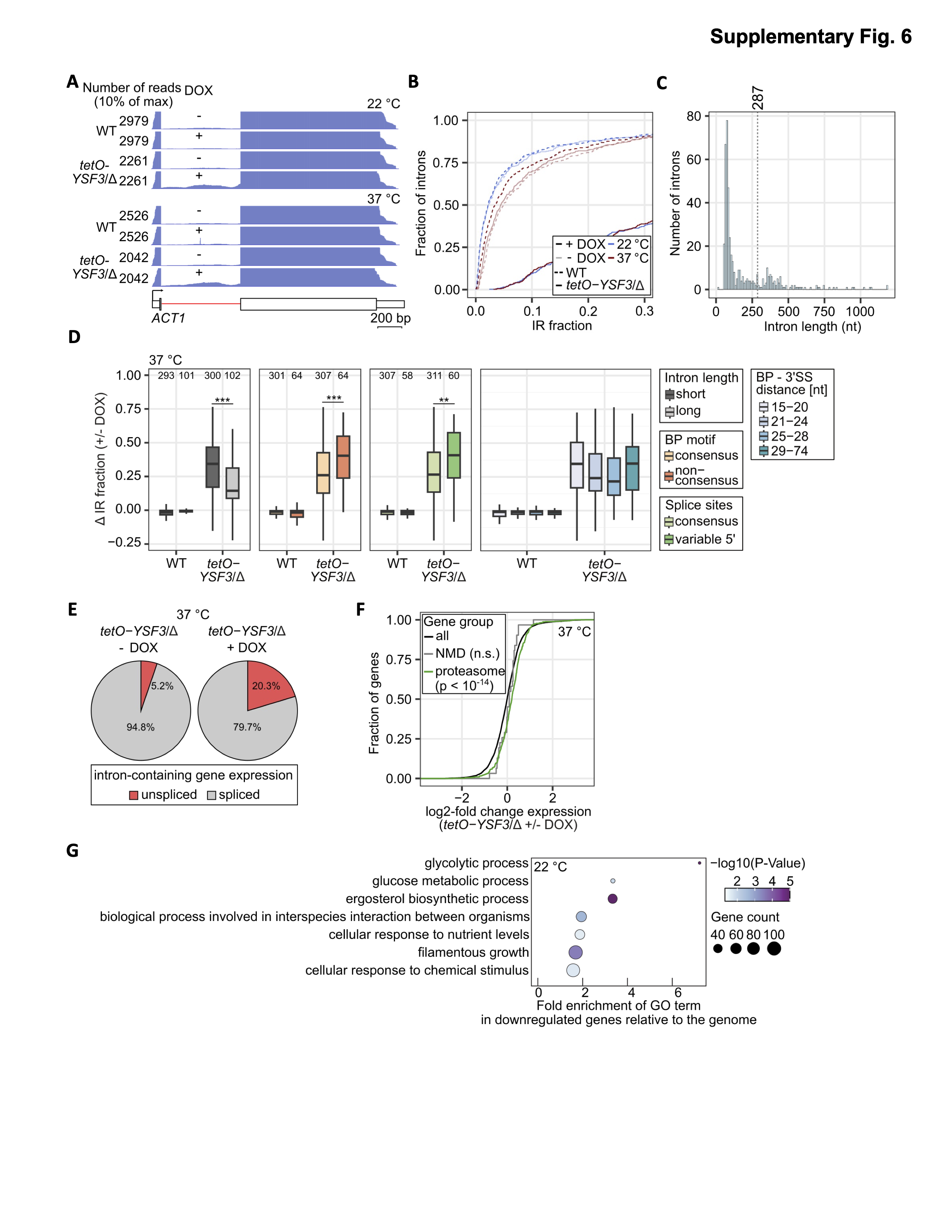

Supplement: S6 Fig — A) RNA-seq read coverage over the ACT1 gene of WT and tetO−YSF3/Δ strains with and without DOX at 22 °C (top) and 37 °C (bottom), complementary to the ACT1 splicing assay (Fig 3C). B) The cumulative distribution shows the IR fraction of 389 introns passing the read count cutoff in all samples. Compared to Fig 3E the x-axis is reduced to 0–0.3 to better visualize differences around the sample cluster that is not YSF3-depleted. The data underlying S6B Fig can be found in S3 Data. C) Bimodal intron length distribution. For analysis of IR dependent on length, we group introns into “short” and “long” based on the minimum at 287 nt (dashed line). The data underlying S6C Fig can be found in S3 Data. D) IR difference of wild type (WT) and tetO-YSF3/Δ strain without and with DOX at 37 °C of introns grouped by their intron length, BP motif, splice site, and BP-3′SS distance, respectively (see Fig 3G for the 22 °C data). Top numbers indicate introns per group. BP-3′SS distance groups have equal bin size. Significances were obtained using Wilcoxon rank-sum test (intron length, BP motif, splice site) and paired Wilcoxon rank-sum test with Benjamini–Hochberg correction (BP–3′SS distance). The data underlying S6D Fig can be found in S4 Data. E) Pie charts of unspliced or spliced intron-containing gene expression in tetO-YSF3/Δ without and with DOX at 37 °C. The data underlying S6E Fig can be found in S5 Data. F) Cumulative distribution of log2-fold change in gene expression between tetO-YSF3/Δ with and without DOX for the indicated gene groups at 37 °C. Significances are relative to the ‘all’ genes (Kolmogorov–Smirnoff test). The data underlying S6F Fig can be found in S5 Data. G) Significantly enriched ‘Biological process’ GO terms among downregulated genes in tetO−YSF3/Δ with DOX at 22 °C. The data underlying S6G Fig can be found in S6 Data. Numerical data underlying this Figure can be found in S1 Data. (TIFF) [file pbio.3003409.s006.tiff]

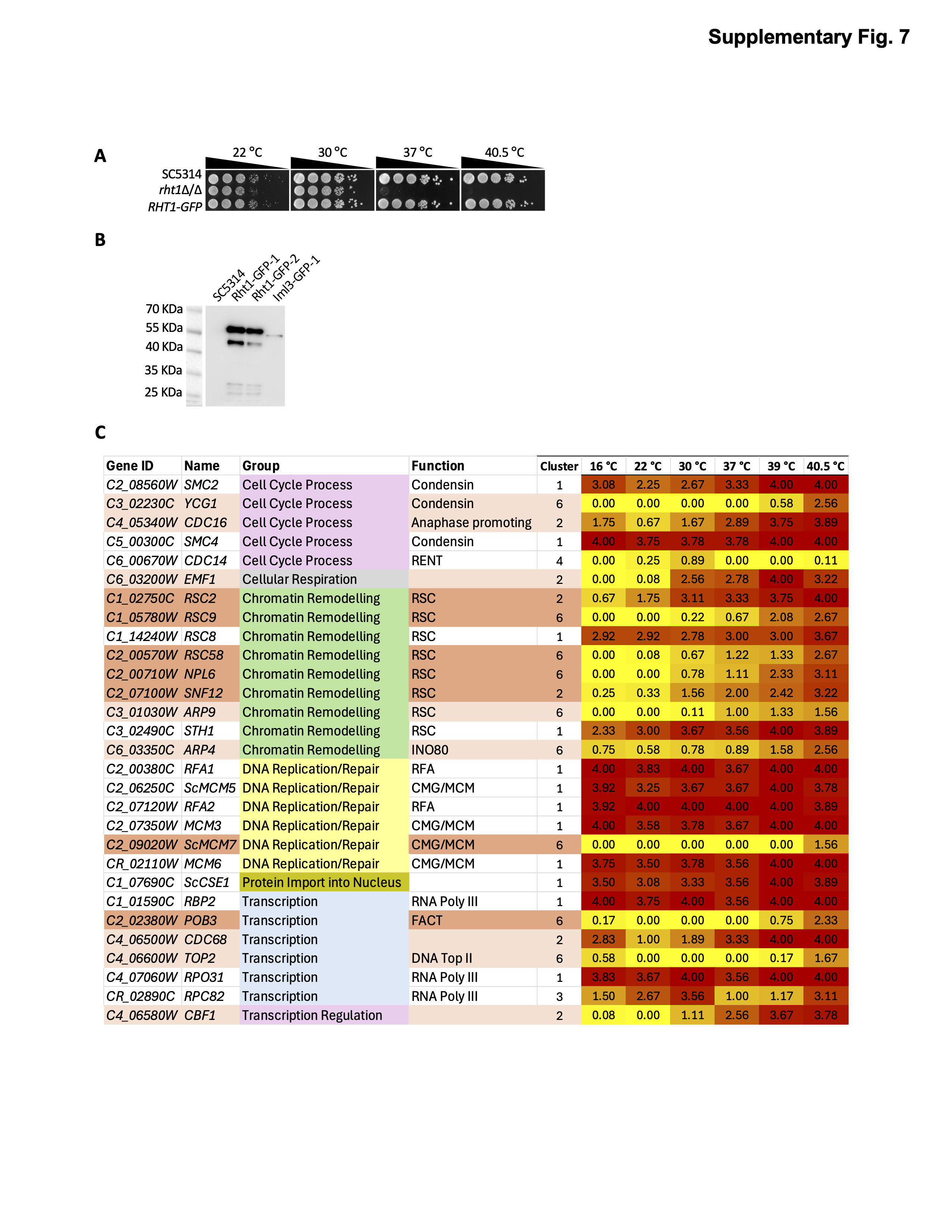

Supplement: S7 Fig — A) To confirm GFP-tagged Rht1 functionality, strains were grown overnight in YPD and spotted in 10-fold dilution starting from an OD600 of 0.8 onto YPD. Plates were incubated at indicated temperatures and imaged after 2 days. B) Detection of GFP-tagged Rht1 by IP-western blot. Wild-type and a GFP-tagged Iml3 strains were used as negative and positive controls. Strains were sub-cultured to an OD600 of 0.2 in YPD and grown at 30 °C for 4 h. Total proteins were extracted. GFP-tagged proteins were purified using anti-GFP magnetic beads, separated on SDS-gel, and blotted for detection of GFP signal with anti-GFP antibody. The Rht1-GFP strain was repeated twice. Full-length Rht1-GFP and Iml3-GFP are predicted to be 57.5and 58.3 kDa in size, respectively. C) Secondary screen results of GRACE strains for Rht1-interacting proteins were analyzed. Gene groups are color-coded as in Fig 5C. DOX scores across the six temperatures are color-scaled as described in Fig 1D. Genes in clusters 2 and 6 are highlighted in orange, with those located on Chromosomes 1 and 2 shown in a darker shade. Source blot images underlying S7B can be found in S1 File Raw_Images. (TIFF) [file pbio.3003409.s007.tiff]

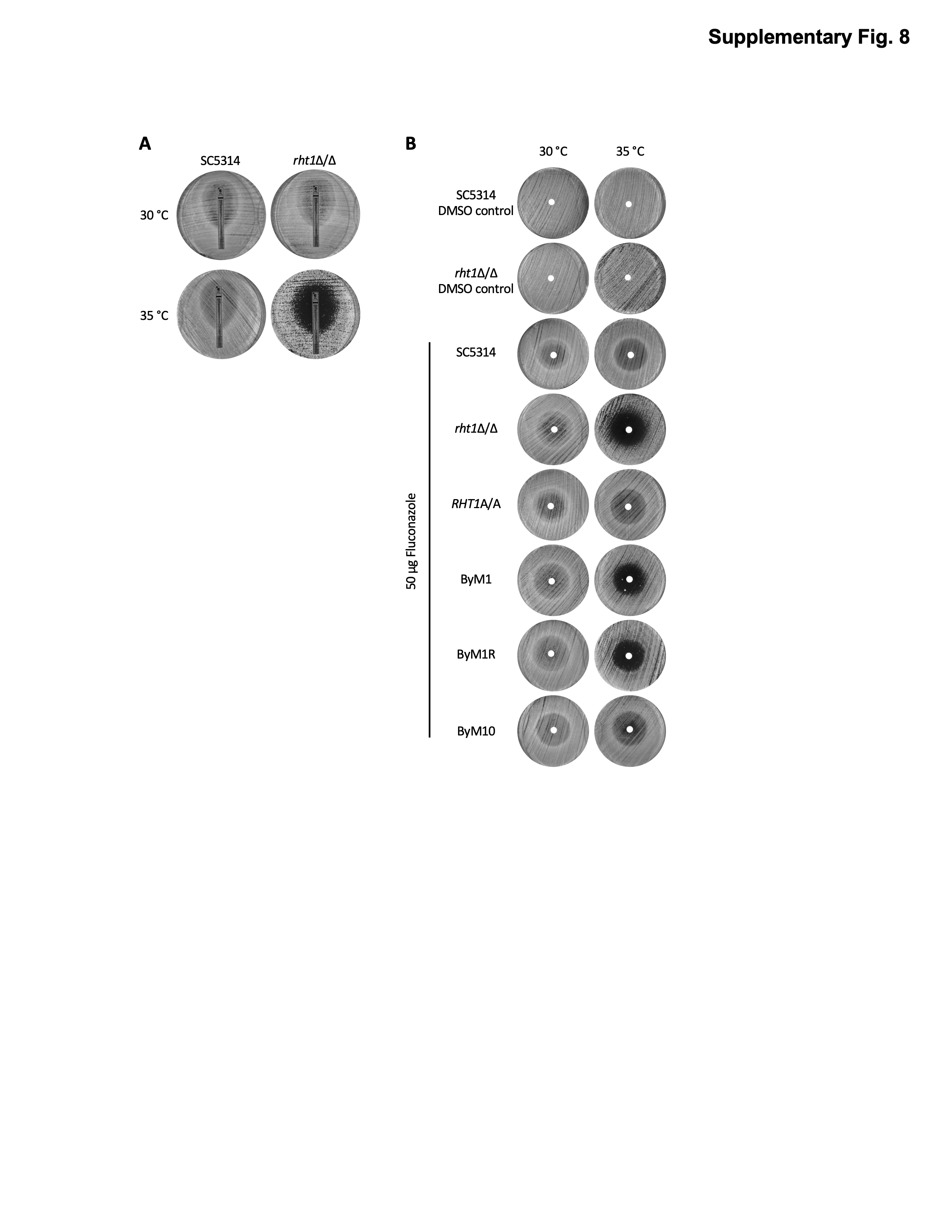

Supplement: S8 Fig — A) Day 5 images for Fig 5F. B) Day 5 images for Fig 5G. (TIFF) [file pbio.3003409.s008.tiff]
