# Supplementary material for: Expansion of the functional genomics GRACE library reveals genes relevant for temperature-dependent fitness in Candida albicans
Source: PLoS Biol. 2025 Oct 17;23(10):e3003409. doi: 10.1371/journal.pbio.3003409 (PMC12533916; doi:10.1371/journal.pbio.3003409)
Supplement: S1 Table — (DOCX) [file pbio.3003409.s009.docx]

**S1 Table: Strains used in this study.**

| **Strain name** | **Description** | **Genotype** | **Parent** | **Source** |
| --- | --- | --- | --- | --- |
| SC5314 | *C. albicans* wild type | Wild type |  | [1] |
| CaLC6106 | *C. albicans* CaSS1 (GRACE library parent) | *ura3*::*imm434*/*ura3*::*imm434 his3*::*hisG*/*his3*::*hisG leu2*::*tetRGAL4AD- URA*/*LEU2* | SC5314 | [2] |
| GRACE strain *GAR1* | *tetO-GAR1*/*gar1*Δ | As CaSS1, *SAT1*::*tetO-GAR1*/*gar1*::*HIS3* | CaLC6106 | [2] |
| GRACE strain *CBF5* | *tetO-CBF5*/*cbf5*Δ | As CaSS1, *SAT1*::*tetO-CBF5*/*cbf5*::*HIS3* | CaLC6106 | [2] |
| GRACE strain *HSP90* | *tetO-HSP90*/*hsp90*Δ | As CaSS1, *SAT1*::*tetO-HSP90*/*hsp90*::*HIS3* | CaLC6106 | [2] |
| GRACE strain *YSF3* | *tetO-YSF3*/*ysf3*Δ | As CaSS1, *SAT1*::*tetO-YSF3*/*ysf3*::*HIS3* | CaLC6106 | This study |
| GRACE strain *PRP19* | *tetO-PRP19*/*prp19*Δ | As CaSS1, *SAT1*::*tetO-PRP19*/*prp19*::*HIS3* | CaLC6106 | [2] |
| GRACE strain *C6_00110C* | *tetO-C6_00110C*/*C6_00110c*Δ | As CaSS1, *SAT1*::*tetO-C6_00110C*/*C6_00110c*::*HIS3* | CaLC6106 | This study |
| CaLC10712 | *gar1*Δ/Δ | *gar1*Δ::*FRT*/*gar1*Δ::*FRT* | SC5314 | This study |
| CaLC10713 | *GAR1A*/*A* | *gar1*::*GAR1A*/*gar1*::*GAR1A* | CaLC10712 | This study |
| CaLC10714 | *gar1*Δ/Δ BM1 | *gar1*Δ::*FRT*/*gar1*Δ::*FRT* | CaLC10712 |  |
| CaLC10720 | *C6_00110c*Δ/Δ | *C6_00110c*Δ::*FRT*/*C6_00110c*Δ::*FRT* | SC5314 | This study |
| CaLC10721 | *C6_00110C_A*/*A* | *C6_00110c*::*C6_00110C_A*/*C6_00110c*::*C6_00110C_A* | CaLC10720 | This study |
| CaLC10722 | *C6_00110c*Δ/Δ BM1 | *C6_00110c*Δ::*FRT*/*C6_00110c*Δ::*FRT* | CaLC10720 | This study |
| CaLC10723 | *C6_00110c*Δ/Δ BM1R | *C6_00110c*Δ::*FRT*/*C6_00110c*Δ::*FRT* | CaLC10720 | This study |
| CaLC10724 | *C6_00110c*Δ/Δ BM2 | *C6_00110c*Δ::*FRT*/*C6_00110c*Δ::*FRT* | CaLC10720 | This study |
| CaLC10725 | *C6_00110c*Δ/Δ BM2R | *C6_00110c*Δ::*FRT*/*C6_00110c*Δ::*FRT* | CaLC10720 | This study |
| CaLC10726 | *C6_00110c*Δ/Δ BM10 | *C6_00110c*Δ::*FRT*/*C6_00110c*Δ::*FRT* | CaLC10720 | This study |
| CaLC10727 | *C6_00110c*Δ/Δ BM3 | *C6_00110c*Δ::*FRT*/*C6_00110c*Δ::*FRT* | CaLC10720 | This study |
| CaLC10728 | *C6_00110c*Δ/Δ BM3R | *C6_00110c*Δ::*FRT*/*C6_00110c*Δ::*FRT* | CaLC10720 | This study |
| CaLC10730 | *C6_00110c*Δ/Δ BM4 | *C6_00110c*Δ::*FRT*/*C6_00110c*Δ::*FRT* | CaLC10720 | This study |
| CaLC10731 | *C6_00110c*Δ/Δ BM4R | *C6_00110c*Δ::*FRT*/*C6_00110c*Δ::*FRT* | CaLC10720 | This study |
| CaLC10732 | *C6_00110c*Δ/Δ BM5 | *C6_00110c*Δ::*FRT*/*C6_00110c*Δ::*FRT* | CaLC10720 | This study |
| CaLC10733 | *C6_00110c*Δ/Δ BM5R | *C6_00110c*Δ::*FRT*/*C6_00110c*Δ::*FRT* | CaLC10720 | This study |
| CaLC10734 | *C6_00110c*Δ/Δ BM6 | *C6_00110c*Δ::*FRT*/*C6_00110c*Δ::*FRT* | CaLC10720 | This study |
| CaLC10735 | *C6_00110c*Δ/Δ BM6R | *C6_00110c*Δ::*FRT*/*C6_00110c*Δ::*FRT* | CaLC10720 | This study |
| CaLC10736 | *C6_00110c*Δ/Δ BM7 | *C6_00110c*Δ::*FRT*/*C6_00110c*Δ::*FRT* | CaLC10720 | This study |
| CaLC10737 | *C6_00110c*Δ/Δ BM7R | *C6_00110c*Δ::*FRT*/*C6_00110c*Δ::*FRT* | CaLC10720 | This study |
| CaLC10738 | *C6_00110c*Δ/Δ BM8 | *C6_00110c*Δ::*FRT*/*C6_00110c*Δ::*FRT* | CaLC10720 | This study |
| CaLC10739 | *C6_00110c*Δ/Δ BM8R | *C6_00110c*Δ::*FRT*/*C6_00110c*Δ::*FRT* | CaLC10720 | This study |
| CaLC10740 | *C6_00110c*Δ/Δ BM9 | *C6_00110c*Δ::*FRT*/*C6_00110c*Δ::*FRT* | CaLC10720 | This study |
| CaLC10741 | *C6_00110c*Δ/Δ BM9R | *C6_00110c*Δ::*FRT*/*C6_00110c*Δ::*FRT* | CaLC10720 | This study |
| CaLC10748 | *C6_00110C-GFP*/*C6_00110C-GFP* | *C6_00110c*::*C6_00110C-GFP*/*C6_00110c*::*C6_00110C-GFP* | SC5314 | This study |
| CaLC10715 | Wild type *KRR1^109GG^* | *krr1^109G^-SAT1*/*krr1^109G^-SAT1* | SC5314 | This study |
| CaLC10716 | *gar1*Δ/Δ BM1 *KRR1^109GG^* | *gar1*Δ::*FRT*/*gar1*Δ::*FRT* BM1 *krr1^109G^-SAT1*/*krr1^109G^-SAT1* | CaLC10714 | This study |
| CaLC10717 | Wild type *KRR1^109AA^* | *krr1^109A^-SAT1*/*krr1^109A^-SAT1* | SC5314 | This study |
| CaLC10718 | *gar1*Δ/Δ *KRR1^109AA^* | *gar1*Δ::*FRT*/*gar1*Δ::*FRT krr1^109A^-SAT1*/*krr1^109A^-SAT1* | CaLC10712 | This study |
| CaLC10742 | Wild type *IML3^200TT^* | *iml3^200T^-SAT1*/*iml3^200T^-SAT1* | SC5314 | This study |
| CaLC10743 | *C6_00110c*Δ/Δ BM10 *IML3^200TT^* | *C6_00110c*Δ::*FRT*/*C6_00110c*Δ::*FRT* BM10 *iml3^200T^-SAT1*/*iml3^200T^-SAT1* | CaLC10726 | This study |
| CaLC10744 | Wild type *IML3^200TC^* | *iml3^200T^-SAT1*/*iml3^200C^-SAT1* | SC5314 | This study |
| CaLC10745 | Wild type *IML3^200CC^* | *iml3^200C^-SAT1*/*iml3^200C^-SAT1* | SC5314 | This study |
| CaLC10746 | *C6_00110c*Δ/Δ *IML3^200CT^* | *C6_00110c*Δ::*FRT*/*C6_00110c*Δ::*FRT iml3^200T^-SAT1*/*iml3^200C^-SAT1* | CaLC10720 | This study |
| CaLC10747 | *C6_00110c*Δ/Δ *IML3^200CC^* | *C6_00110c*Δ::*FRT*/*C6_00110c*Δ::*FRT iml3^200C^-SAT1*/*iml3^200C^-SAT1* | CaLC10720 | This study |
| All other strains used in this manuscript are members of the GRACEv1, GRACEv2, and GRACEv3 collections | | | | |

**References**

1. Odds FC. Effects of temperature on anti-Candida activities of antifungal antibiotics. Antimicrob Agents Chemother. 1993;37(4):685-91. doi: 10.1128/AAC.37.4.685. PubMed PMID: 8494363; PubMed Central PMCID: PMCPMC187735.

2. Roemer T, Jiang B, Davison J, Ketela T, Veillette K, Breton A, et al. Large-scale essential gene identification in *Candida albicans* and applications to antifungal drug discovery. Mol Microbiol. 2003;50(1):167-81. Epub 2003/09/26. doi: 10.1046/j.1365-2958.2003.03697.x. PubMed PMID: 14507372.
