# Supplementary material for: Expansion of the functional genomics GRACE library reveals genes relevant for temperature-dependent fitness in Candida albicans
Source: PLoS Biol. 2025 Oct 17;23(10):e3003409. doi: 10.1371/journal.pbio.3003409 (PMC12533916; doi:10.1371/journal.pbio.3003409)
Supplement: S2 Table — (DOCX) [file pbio.3003409.s010.docx]

**S2 Table: Plasmids used in this study.**

| **Plasmid name** | **Description** | **Source** |
| --- | --- | --- |
| pLC763 | pCa-FLP-NAT-*tetO* | [1] |
| pLC1251 | pUC19+*HIS3* | [2] |
| pLC49 | FLP-SAT1 | [3] |
| pLC1081 | pV1093(CaCAS9&sgRNA) | [4] |
| pLC2052 | pCaGAR1 | This study |
| pLC2053 | pCaKRR1-109G | This study |
| pLC2054 | pCaKRR1-109A | This study |
| pLC2055 | pCaIML3-200T | This study |
| pLC2056 | pCaIML3-200C | This study |

**References**

1. Gerami-Nejad M, Berman J, Gale CA. Cassettes for PCR-mediated construction of green, yellow, and cyan fluorescent protein fusions in *Candida albicans*. Yeast. 2001;18(9):859-64. doi: 10.1002/yea.738. PubMed PMID: 11427968.

2. Fu C, Zhang X, Veri AO, Iyer KR, Lash E, Xue A, et al. Leveraging machine learning essentiality predictions and chemogenomic interactions to identify antifungal targets. Nat Commun. 2021;12(1):6497. Epub 20211111. doi: 10.1038/s41467-021-26850-3. PubMed PMID: 34764269; PubMed Central PMCID: PMCPMC8586148.

3. Morschhauser J, Michel S, Staib P. Sequential gene disruption in *Candida albicans* by FLP-mediated site-specific recombination. Mol Microbiol. 1999;32(3):547-56. doi: 10.1046/j.1365-2958.1999.01393.x. PubMed PMID: 10320577.

4. Min K, Ichikawa Y, Woolford CA, Mitchell AP. *Candida albicans* gene deletion with a transient CRISPR-Cas9 system. mSphere. 2016;1(3). Epub 20160615. doi: 10.1128/mSphere.00130-16. PubMed PMID: 27340698; PubMed Central PMCID: PMCPMC4911798.
