# Supplementary material for: Expansion of the functional genomics GRACE library reveals genes relevant for temperature-dependent fitness in Candida albicans
Source: PLoS Biol. 2025 Oct 17;23(10):e3003409. doi: 10.1371/journal.pbio.3003409 (PMC12533916; doi:10.1371/journal.pbio.3003409)
Supplement: S3 Table — Verified variants for gar1Δ/ΔByM1 and rht1Δ/ΔByM10 were provided in the table. (DOCX) [file pbio.3003409.s011.docx]

**S3 Table: Whole-genome sequencing variant calls.**

| **Mutant background** | **Lineage** | **Gene** | **Amino acid substitution** | **Type of mutation** |
| --- | --- | --- | --- | --- |
| *gar1*Δ/Δ | ByM1 | *KRR1* | Glu37Lys | LOH |
| *C6_00110c*Δ/Δ | ByM10 | *IML3* | Leu67Ser | SNP |
