# Supplementary material for: Expansion of the functional genomics GRACE library reveals genes relevant for temperature-dependent fitness in Candida albicans
Source: PLoS Biol. 2025 Oct 17;23(10):e3003409. doi: 10.1371/journal.pbio.3003409 (PMC12533916; doi:10.1371/journal.pbio.3003409)

Raw gel images for Figure 3C: the top lane displays three biological replicates corresponding to the result shown in Figure 3C, while the bottom lane shows the same PCR performed using water in place of cDNA as a negative control. The middle panels from the second biological replicate are presented in Figure 3C.

22 °C

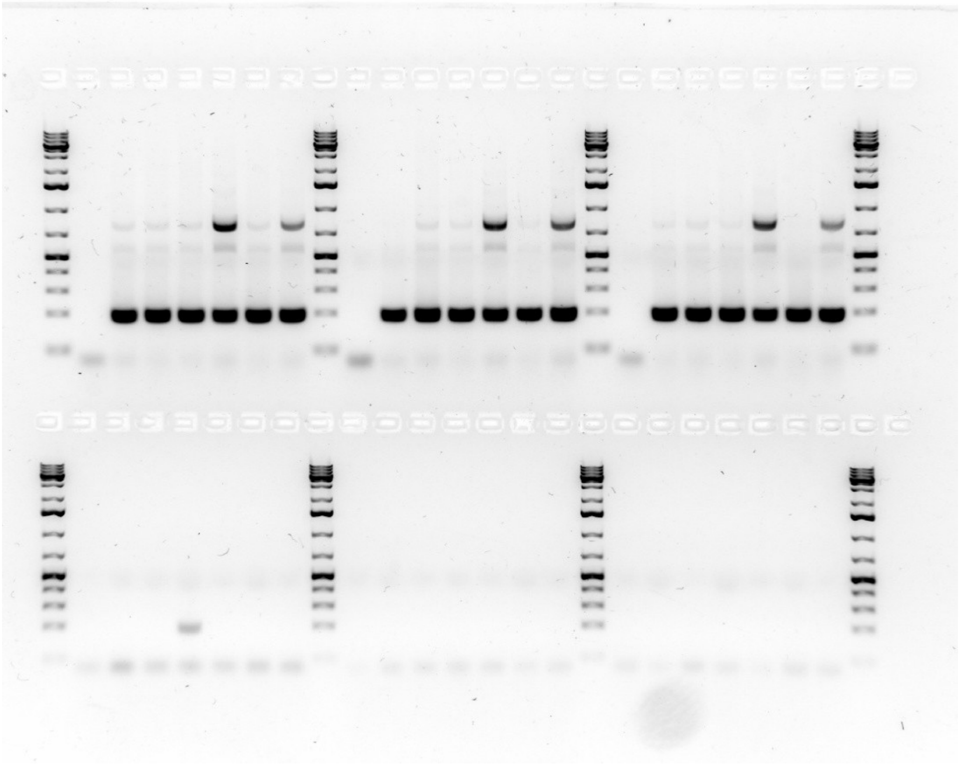

37 °C

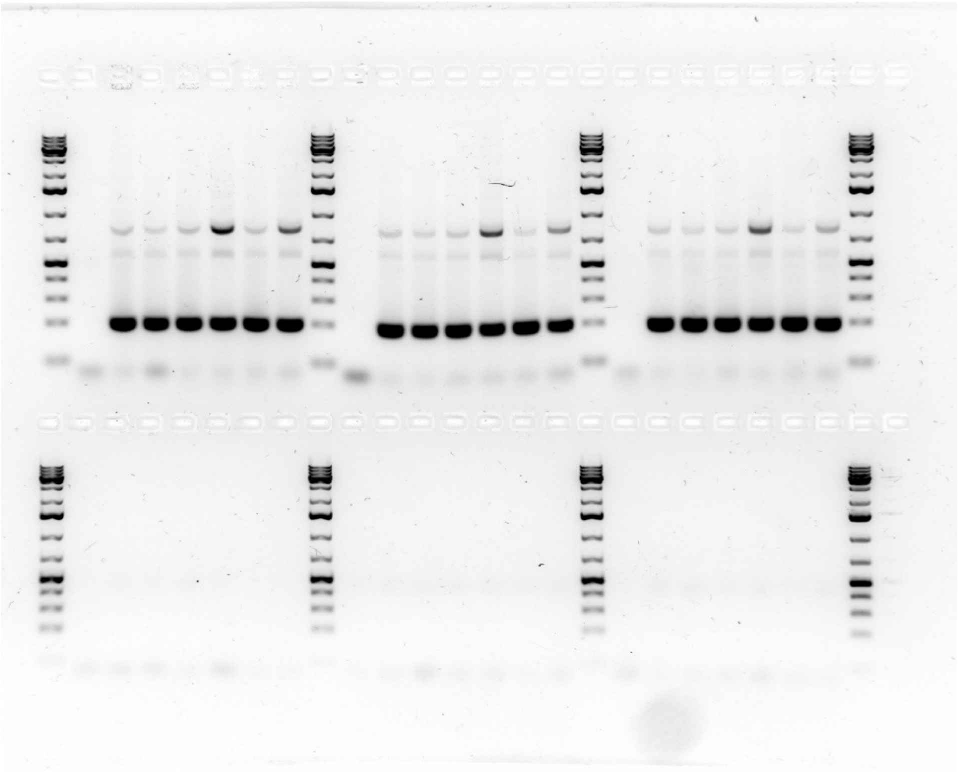

Raw blot images for Figure S7B: the ladder lane was cropped from the colorimetric ladder image, and the chemiluminescence blot image was flipped to match the order shown in Figure S7B.

Colorimetric ladder image

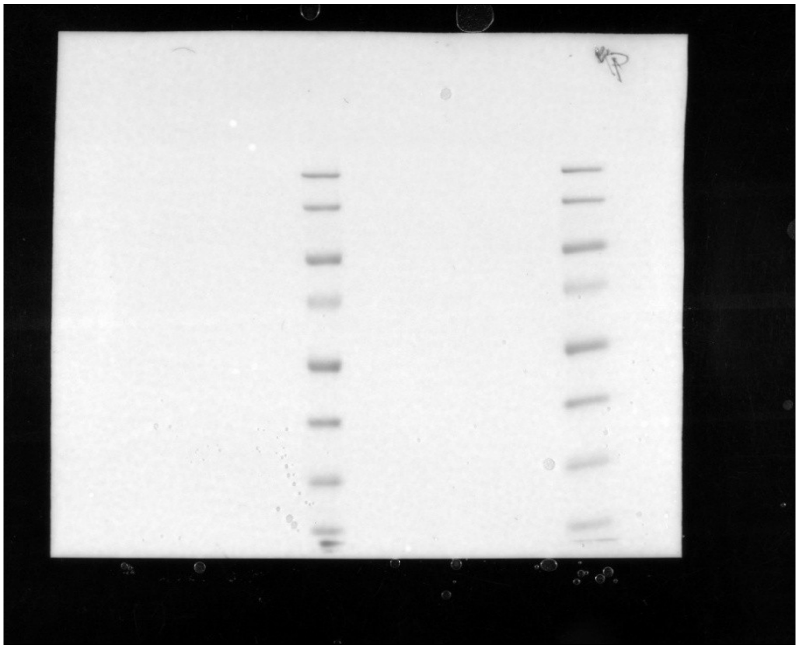

Chemiluminescence blot

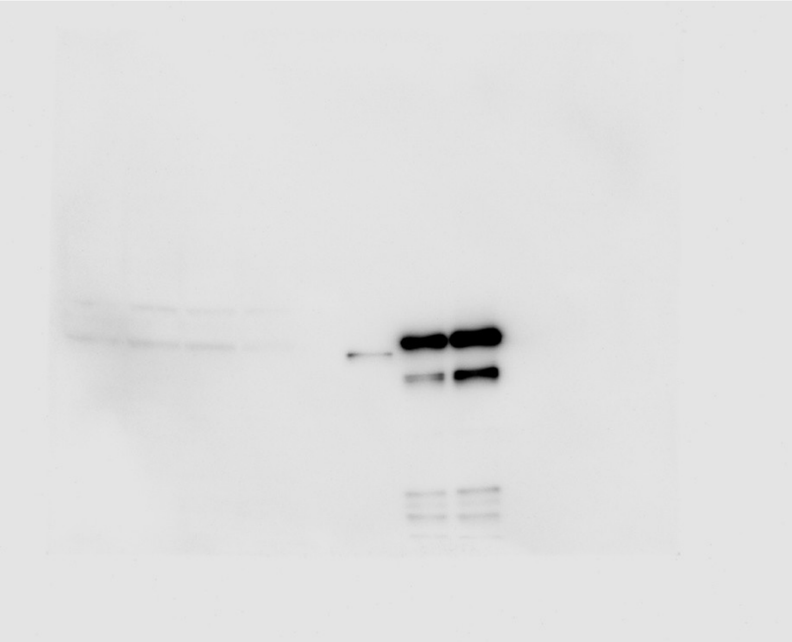

Supplement: S1 File — (PDF) [file pbio.3003409.s020.pdf]
